# Analysis of a novel phage as a promising biological agent targeting multidrug resistant *Klebsiella pneumoniae*

**DOI:** 10.1186/s13568-025-01846-0

**Published:** 2025-03-05

**Authors:** Mahmoud A. Abdel-Razek, Shaimaa I. Nazeih, Nehal Yousef, Momen Askoura

**Affiliations:** Department of Microbiology and Immunology, Faculty of Pharmacy, Zagazig University, Zagazig, 45519 Egypt

**Keywords:** *Klebsiella pneumoniae*, Phage therapy, Multidrug resistance, Bacteriophage, Biofilm, Genomic characterization

## Abstract

The rise of deaths by resistant bacteria is a global threat to public health systems. *Klebsiella pneumoniae* is a virulent pathogen that causes serious nosocomial infections. The major obstacle to bacterial treatment is antibiotic resistance, which necessitates the introducing of alternative therapies. Phage therapy has been regarded as a promising avenue to fight multidrug-resistant (MDR) pathogens. In the current study, a novel phage vB_KpnP_KP17 was isolated from sewage, and its lytic potential was investigated against *K. pneumoniae*. The isolated phage vB_KpnP_kP17 was lytic to 17.5% of tested *K. pneumoniae* isolates. One step growth curve indicated a virulent phage with a short latent period (20 min) and large burst size (331 PFU/cell). Additionally, vB_KpnP_kP17 maintained its activity against planktonic cells over a wide range of pH, temperature and UV irradiation intervals. The potential of vB_KpnP_KP17 as antibiofilm agent was revealed by the biofilm inhibition assay. The isolated phage vB_KpnP_KP17 at multiplicity of infection (MOI) of 10 inhibited more than 50% of attached biofilms of tested *K. pneumoniae* isolates. The genome of vB_KpnP_kP17 was characterized and found to be a linear dsDNA of 39,936 bp in length and GC content of 52.85%. Additionally, the absence of toxicity, virulence and antibiotic resistance genes further confirms the safety of vB_KpnP_KP17 for clinical applications. These characteristics make vB_KpnP_KP17 of a potential therapeutic value to manage MDR *K. pneumoniae* infections. Additionally, the formulation of vB_KpnP_KP17 in a cocktail with other lytic phages or with antibiotics could be applied to further limit biofilm-producing *K. pneumoniae* infections.

**Supplementary Information:**

The online version contains supplementary material available at 10.1186/s13568-025-01846-0.

## Introduction

*Klebsiella pneumoniae* (*K. pneumoniae*) is an opportunistic pathogen that colonizes the mucus membranes of many organs commensally (Asif et al. 2023). *K. pneumoniae* turns into pathogenic in immunocompromised patients and causes multiple infections such as pneumonia, liver abscess, septicemia, wound infection and urinary tract infection (Townsend et al. 2020). *K. pneumoniae* is one of the most isolated nosocomial pathogens and represents 33% of Gram-negative bacterial infections (Navon-Venezia et al. 2017). Seriously, *K. pneumoniae* belongs to the ESKAPE group (*Enterococcus faecium*, *Staphylococcus aureus*, *Klebsiella pneumoniae*, *Acinetobacter baumannii*, *Pseudomonas aeruginosa* and *Enterobacter species*) and represents a global threat by developing the multidrug resistance (MDR). The WHO organization warned that 10 million people would be killed by resistant microbes by 2050 (Alrafaie and Stafford 2023; Liang et al. 2023). The pathogenicity of *K. pneumoniae* is attributed to many virulence factors including capsular polysaccharide, siderophores and adherence proteins of fimbriae (Effah et al. 2020). Capsular polysaccharide (K antigen) is a major virulence determinant in *K. pneumoniae* that hinders the penetration of antibiotics and represents a mask structure for bacteria to prevent opsonization by immune cells. Additionally, capsules enhance the chronicity of bacterial infections through biofilm synthesis (Beamud et al. 2023; Eckstein et al. 2021; Song et al. 2021). Interestingly, over 130 capsular serotypes have been characterized; the K2 capsule type is the most abundant in highly virulent strains of *Klebsiella pneumoniae* (Volozhantsev et al. 2022). Another important virulent factor in *K. pneumoniae* is the higher capability to form biofilm. Biofilm is a three-dimensional network of polysaccharides, proteins, and lipids that interconnect with nucleic acids. This polymeric mixture is a protective barrier for *K. pneumoniae* against antibiotics, immune cells, heavy metals and ultraviolet radiation. Moreover, the biofilm provides nutritious elements to the hidden viable bacteria (Flemming and Wingender 2010). The chronicity of infection is triggered by biofilm formation, which blocks the entry of antibiotics into microbial cells (Asghar et al. 2022).

The resistance developed by nosocomial pathogens has led to failure of current antibiotics and high mortality rates (Magill et al. 2014). Different mechanisms have been developed by *K. pneumoniae* to resist the antimicrobial agents. These resistance strategies include: synthesis of degrading enzymes, change in the conformation of active sites, multiplication of the target site, and preventing the intracellular accumulation of antibiotics by decreasing the influx or increasing expulsion through the efflux pump (Abdel-Halim et al. 2022). The resistance of bacterial strains to the final-line antibiotics has markedly increased (Årdal et al. 2020; Balcão et al. 2022). Carbapenems are among the last resorts of antimicrobial agents that have been prescribed against resistant bacteria. Unfortunately, carbapenem-resistant *K. pneumoniae* (CRKP) is currently an annoying pathogen at hospitals around the world (M Li et al. 2020). Therefore, various strategies rather than antibiotics such as monoclonal antibodies and phage therapy have been developed for treatment of resistant pathogens (Criscuolo et al. 2017).

Bacteriophage (phage) is a virus that commands the genetic material of bacteria and new viral progenies are released to kill more bacterial cells (Henry and Debarbieux 2012). A list of advantages has been achieved by phage therapy over antibiotics, such as host specificity, self-replication, lack of inducing resistance, and no adverse action on normal flora (Loc-Carrillo and Abedon 2011). Moreover, phages secrete polysaccharide depolymerase enzymes which play a role in biofilm clearance (Volozhantsev et al. 2022). In combination with antibiotics, phages increase the sensitivity of bacteria toward antibiotics without cross-resistance (Chan et al. 2016). Phages in various formulations were tested for therapeutic applications. Preclinical studies on mice models had been conducted to cure various diseases such as pneumonia, psoriasis, burn wounds, and diabetic foot ulcer (Palaniappan and Dayanithi 2021). Therefore, phage formulations are now developed by pharmaceutical industries to treat MDR bacterial infection (Merabishvili et al. 2019).

The aim of current study is to isolate a virulent phage targeting MDR *K. pneumoniae* isolates from various clinical sources. The isolated phage will be characterized morphologically by transmission electron microscopy (TEM) and genetically by whole genome sequencing. Moreover, the biological characterization of isolated phage including host range, efficiency of plating (EOP), one-step growth curve, in vitro killing assay, and biofilm clearance will be conducted to prove the therapeutic efficacy of isolated phage.

## Materials and methods

### Isolation and identification of *K. pneumoniae*

A total of 39 *K. pneumoniae* isolates were obtained from clinical laboratories of Zagazig University Hospital, Zagazig, Egypt. *K. pneumoniae* isolates were aseptically collected from different sources including feces (n = 2), sputum (n = 9), pus (n = 8), urine (n = 11) and blood (n = 9), without direct contact with patients. The reference strain *K. pneumoniae* ATCC700603 was also included in the present study. Pure colonies of *K. pneumoniae* isolates were obtained by subculture on tryptone soy agar plates (TSA, Oxoid, UK) and were further identified by the VITEK MS identification system. Additionally, the isolates were biochemically confirmed as *K. pneumoniae* according to Hansen et al. (Hansen et al. 2004). Bacterial isolates were preserved in TS broth containing 25% glycerol and kept at—80 °C.

### Antibiotic susceptibility testing

The susceptibility of *K. pneumoniae* clinical isolates to various antibiotics including ceftriaxone (CRO, 30 µg), cefepime (FEP, 30 µg), piperacillin-tazobactam (TZP, 110/10 µg), meropenem (MEM, 10 µg), azithromycin (AZM, 15 µg), chloramphenicol (C, 30 µg), amikacin (AK, 30 µg), tetracycline (TE, 30 µg), tigecycline (TGC, 15 µg), trimethoprim-sulfamethoxazole (SXT, 1.25/23.75 µg), levofloxacin (LEV, 5 µg), and ofloxacin (OFX, 5 µg) was checked by the disk diffusion method. Briefly, bacterial isolates were cultured overnight on TS agar plates. Separate colonies were suspended in phosphate buffered saline (PBS) with turbidity adjusted to 0.5% McFarland. Sterile swap was dipped into saline suspension and spread uniformly to the surface of Muller-Hinton (MH) agar plates. Antibiotic disks were aseptically placed on the agar surface followed by incubation of plates at 37 °C for 24 h. The diameter of clear zone around disks was recorded. The resistance profile of *K. pneumoniae* was interpreted as resistant, intermediate, and sensitive according to the Clinical and Laboratory Standards Institute (CLSI) recommendations (Lewis and James 2022). Bacterial multidrug resistance (MDR) was determined as bacterial resistance to at least one antibiotic in three or more antibiotic classes. Multiple antibiotic resistance (MAR) was justified to calculate the modified index (m-MAR) to account for the intermediately susceptible isolates. The m-MAR index is the ratio between the number of antibiotics to which a bacterial isolate is resistant and intermediate to the total number of tested antibiotics. Scores have been assigned to calculate the m-MAR index as resistant equal 1, intermediate as 0.5, and sensitive as zero (Zaki et al. 2023).

### Isolation of phage

Sewage samples were collected from different regions in Zagazig, Egypt. The initial purification procedure was performed on sewage samples to remove cellular debris by centrifugation at 6000 × *g* for 15 min followed by filtration using a 0.22 µm filter. The phage was isolated according to enrichment protocol (Wintachai et al. 2019). In brief, the treated sample was mixed with a double concentrated TS broth (2 × TS broth) and forty isolates of exponentially growing cultures in a large flask. The bulk culture was incubated overnight at 37 °C. Afterwards, the incubated culture was centrifuged at 6000 × *g* for 15 min and bacterial pellet was discarded. The supernatant was added to chloroform (9:1 v/v) in a sterile 15 mL centrifuge tube. The activity of isolated phage was primary screened on *K. pneumoniae* by the spot test (Pereira et al. 2011). Briefly, 100 µL of bacteria was mixed with 4 mL of soft agar (0.6% agar), and the mixture was spread onto the surface of bottom agar (1.5% agar). The supernatant was pipetted onto each bacterial lawn to detect the presence of phage as a clear zone on a turbid lawn. The clear zone was aseptically picked up into a sterile 2 mL centrifuge tube for subsequent purification procedure.

### Purification and propagation of isolated phage

The phage lysate was purified by the double-overlay plaque method (Santos et al. 2009). In brief, the initial phage stock was tenfold serially diluted in saline-magnesium (SM) buffer [8 mM MgSO_4_, 100 mM NaCl, 0.01% gelatin, 50 mM Tris HCl (pH 7.4)], and the phage dilution was incubated in equal volume (200 µL) with the bacterial host. The infected bacterial culture (400 µL) was mixed with 4 mL of soft agar and the final bulk was uniformly distributed into the surface of bottom agar layer. The procedure was repeated three times, where similar plaques resulted from the double-overlay plaque assay were picked up in a sterile SM buffer. The propagation of phage was carried out on plates of confluent lysis, where the plates were soaked by 4 mL of SM buffer and preserved at 4 °C. After 12 h, the soft agar layer and the buffer mixture were scraped off by a sterile spreader. The resulting suspension was aseptically collected and centrifuged at 6000 × *g* for 15 min and the supernatant was filtered to obtain a high-titer phage lysate (Anany et al. 2018)**.** The purified solution of isolated phage was stored at 4 °C for subsequent experiments. The isolated bacteriophage vB_KpnP_KP17 is stored in the Department of Microbiology and Immunology, Faculty of Pharmacy, Zagazig University and is available upon request by contacting the corresponding author; Momen Askoura (Momenaskora@yahoo.com).

### Determination of phage host range

The spot assay method was employed to determine the virulence of isolated phage against 40 *K. pneumoniae* isolates and the reference strain *K. pneumoniae* ATCC 700603. In addition, the lytic spectrum of isolated phage was screened against other bacterial species including *Escherichia coli* ATCC 10536, ATCC O26, ATCC O78 and O157, *Pseudomonas aeruginosa* ATCC 27853, ATCC 9027 and PAO1, *Staphylococcus aureus* ATCC 6538 and ATCC 9295, as well as *Serratia marcescens*, *Salmonella* Typhimurium, and *Bacillus cereus* clinical isolates. Plates of the spot assay were checked for lysis zones after incubation for 24 h.

### Efficiency of plating (EOP)

The killing efficiency of isolated phage on host bacteria was compared to other sensitive isolates using the double-overlay plaque assay. EOP is defined as the average number of plaque-forming units (PFU/mL) on a sensitive strain/average (PFU/mL) on the host strain. The estimates of EOP were classified into four classes as follows: high productive (≥ 0.5), moderate (0.5–0.1), low (0.1–0.001), and inefficient (≤ 0.001) (Khan Mirzaei and Nilsson 2015).

### Transmission electron microscope (TEM)

A drop of high titer phage lysate (10^14^ PFU/mL) was loaded onto a carbon-coated copper grid and negatively stained by phosphotungstic acid. The bacteriophage was morphologically investigated by TEM (Hitachi H600A, Japan). The photographed virion was classified following the guidelines of the International Committee on Taxonomy of Viruses (ICTV) (Walker et al. 2022)**.**

### Physicochemical stability of phage

The thermotolerance ability of isolated phage was measured at various temperatures (30–100 °C) for 1 h. Regarding pH stability, the isolated phage was incubated for 1 h in SM buffer at different pH values range from 1 to 14 adjusted by 1M HCl and 1 M NaOH. Considering the chloroform tolerance, the solution of phage in SM buffer was incubated at different concentrations of chloroform (10, 30, 50, 70, 90, and 100%) for 1 h with continuous shaking. To check the ultraviolet stability, the phage lysate in a sterile petri dish was exposed to UV light at variable irradiation times (0, 15, 30, 45, 60, 75, and 90 min). The phage titer was enumerated by the double-overlay plaque method following exposure to previous conditions in order to determine phage stability (Wen et al. 2022)**.**

### One-step growth phase

The host bacteria at an optical density (OD) of 0.2 (early log phase) was infected by isolated phage at MOI of 0.1 for 5 min at 37 °C. This mixture was centrifuged at 6000 × *g* for 10 min and the pellet was resuspended in 10 mL TS broth. A volume of 100 µL of suspended phage was mixed with fresh exponentially grown host bacteria to perform double-overlay plaque assay every 10 min interval from 0 to 80 min. Finally, the phage latent period and burst size were determined (Abdelghafar et al. 2023). The latent period was determined as the duration from free phage adsorption to the release of new progeny after bacteriolysis. The phage burst size was calculated as the ratio of phage titer expressed PFU/mL at the plateau phase to its titer at latency phase (Abedon 2023).

### Time killing assay

In vitro bacteriolytic analysis was performed to determine the sensitivity of planktonic cells to the isolated phage. Suspension of host bacteria (OD = 0.2) was challenged by isolated phage at different MOIs (0.1, 1 and 10) in separate 15 mL centrifuge tubes. Three tubes were assigned for each MOI and the untreated bacteria were marked as a positive control. The change in bacterial turbidity was measured spectrophotometrically at 570 nm in a 96-well microtiter plate (Costar™; Corning™) every 1 h for 12 h. The assay was carried out three times, and results were calculated as means ± standard (Sattar et al. 2023).

### Biofilm assay

Biofilm-forming capacity was examined for all bacterial isolates by the crystal violet technique (Stepanović et al. 2007). Overnight *K*. *pneumoniae* culture was adjusted to the turbidity of 0.5% McFarland and then diluted in double-strength TS broth at a ratio of 1:100. A volume of 200 µL of the diluted bacterial culture was transferred into each well of 96-well plate followed by incubation at 37 °C for 24 h. The planktonic cells were discarded, and wells were washed with PBS and dried. The biofilm matrix was fixed by absolute methanol and stained with 1% crystal violet. The adhered dye was solubilized by 33% glacial acetic acid, and the absorbance was measured spectrophotometrically at 570 nm (Bio-Tek Synergy HT microplate reader, USA).

### Biofilm inhibition and degradation assay

Considering biofilm inhibition, the isolated phage was mixed with the host bacteria at various MOIs (0.1, 1 and 10) in a 96-well microtiter plate. The inoculated plate was incubated for 12 h at 37 °C. Afterwards, the microtiter plate was washed with PBS, and formed biofilm was fixed with methanol. The crystal violet technique was performed to stain and solubilize the biofilm. Finally, the optical density at 570 nm was recorded by the microtiter plate reader. Regarding biofilm degradation, the mature biofilms of tested bacterial isolates were allowed to form by inoculating bacterial culture at bacterial density of 10^6^ CFU/mL in a 96-well microtiter plate, and the plate was incubated overnight at 37 °C. The plate was washed by PBS three times to discard planktonic cells. The isolated phage was applied at various titers of 10^5^, 10^6^ and 10^7^ PFU/mL to break the bound biofilm and incubated for 12 h at 37 °C. The attached biofilm was quantified by the crystal violet assay exactly as described above (Li et al. 2023).

### Phage DNA extraction and whole genome sequencing

The extraction of genomic DNA from pure phage lysate was performed by the QIA amp® DNA Mini Kit (QIAGEN, Germany) following the manufacturer’s instructions. The extracted DNA was evaluated by a Qubit fluorometer and Nanodrop spectrophotometer to ensure the quantity and quality of the sample. A library rapid barcoding kit was obtained and genomic DNA was cleaved by transposomes into fragments with barcoded tags. Sample was loaded on flow cell to run whole genome sequencing using the Oxford Nanopore technique on MinION Mk1C. Finally, the annotated genome of isolated phage vB_KpnP_KP17 was deposited in the database of NCBI GenBank under the accession number PP096838.

### Bioinformatic analysis

The quality of raw data of the resulted sequences was checked by FASTQC (Brown et al. 2017). Low-quality nucleotides, especially adaptor sequences, were trimmed using Trimmomatic v0.36 (Bolger et al. 2014). Short and long reads were combined into contigs by Unicycler v0.4.8 (Wick et al. 2017). Assembly quality was evaluated by QUAST v 5.0.2 to generate a single contig (Gurevich et al. 2013). The potential functions of open reading frames (ORFs) in the phage genome were analyzed by Prokka v 1.14 (Seemann 2014). Additionally, further annotation was performed using InterPro tool to assign potential roles for hypothetical proteins (https://www.ebi.ac.uk/interpro/). The genomic features based on gene function were visually represented in a circular map via CGView (Grant et al. 2012). The whole ORFs were examined to find out putative tRNA genes using tRNAscan-SE 1.21 (Lowe and Chan 2016). The coding sequences (CDS) in the phage genome were checked for resistance and virulence genes using the databases of Antibiotic Resistant Genes, ResFinder, and Virulence factor (Alcock et al. 2020; Kleinheinz et al. 2014). The most closely matched phages were determined for phylogeny by comparing the nucleotide sequence of isolated phage genome against other phages in GenBank of the NCBI database. Alignment of conserved genomic regions in terms of gene loss, gain and rearrangements was performed by Multiple Alignment of Conserved Genomic Sequence With Rearrangements (MAUVE) (Darling et al. 2004). Comparison of the isolated phage genome with its close homologs was visualized by the Easyfig program (Sullivan et al. 2011). Additionally, these phages were compared based on all shared proteins by the proteome comparison tool of PathoSystems Resource Integration Center (PATRIC) (Overbeek et al. 2014). The intergenomic similarity between the isolated phage and more distantly phages was calculated using VIRDIC (Virus Intergenomic Distance Calculator) (Moraru et al. 2020). The classification based on gene-sharing networks was performed by Viral CONTigs Automatic Clustering (vConTACT2) and visualized by Cytoscape (Bin Jang et al. 2019; Shannon et al. 2003). The intergenomic distances between phages were computed by Virus Classification and Tree Building Online Resource (VICTOR) to construct evolution trees (Meier-Kolthoff and Göker 2017). Proteome-based clustering by VIPTree was constructed based on the tBLASTx analysis (Nishimura et al. 2017). The Molecular Evolutionary Genetics Analysis (MEGA) software was conducted to construct phylogenetic trees based on the whole genome and conserved genes encoding major capsid protein, terminase large subunit, and RNA polymerase (Kumar et al. 2016).

### Statistical analysis

Statistical analysis was carried out by GraphPad Prism 9 software using Student *t*-test or one-way ANOVA. All procedures were performed in triplicate, and data were interpreted as the mean ± standard errors.

## Results

### Bacterial identification and antimicrobial sensitivity testing

**Fig. 1:**
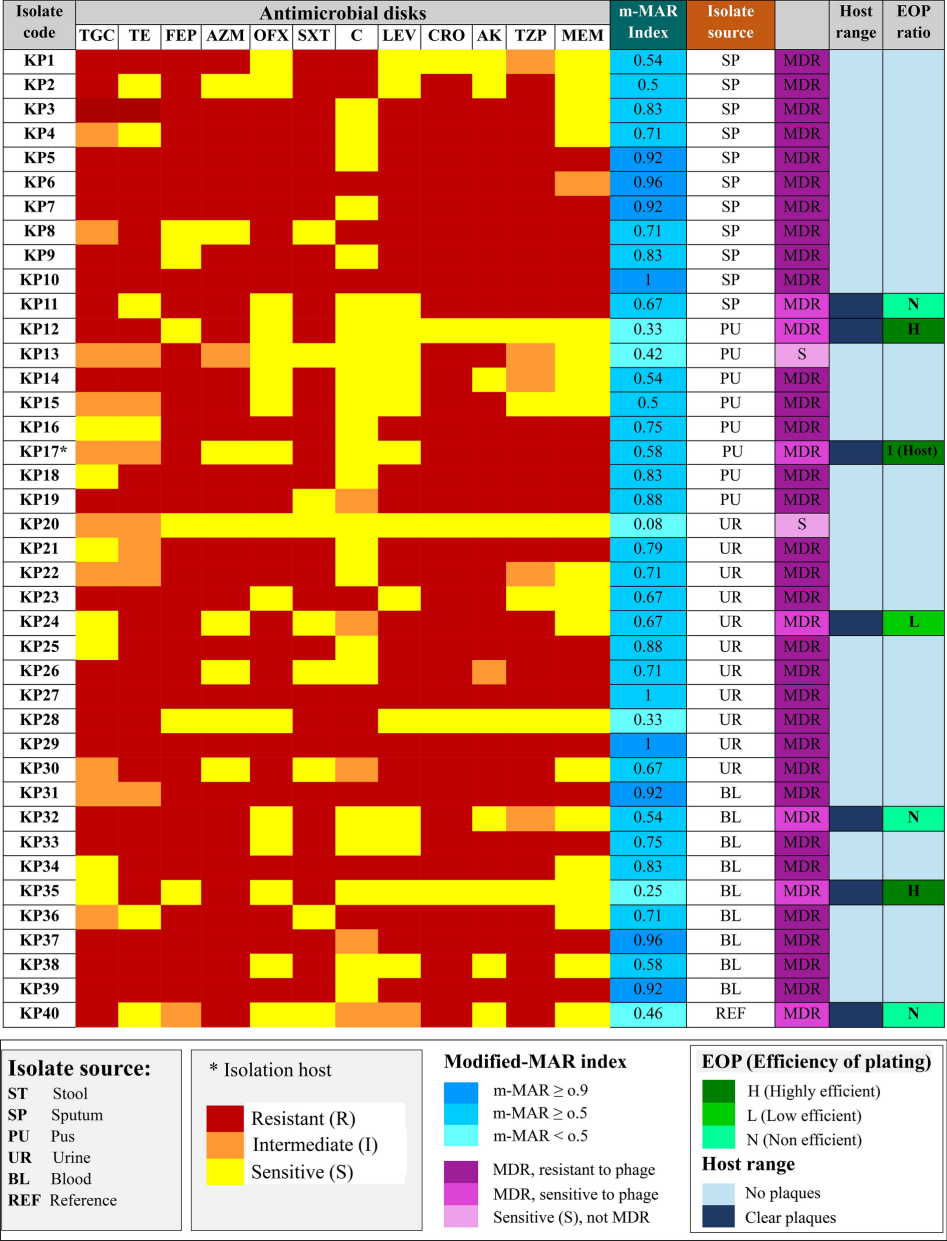
Antibiotic sensitivity profile of *K. pneumoniae* clinical isolates. The susceptibility profile of *K. pneumoniae* isolates against antibiotics from different antibiotic classes is represented as a heatmap. Additionally, the MDR profile, modified MAR index, host range and susceptibility of *K. pneumoniae* isolates to vB_KpnP_KP17 are indicated. The efficiency of plating (EOP) was evaluated according to the degree of productivity as H (highly), L (low) and N (not) efficient

A total of 40 *K*. *pneumoniae* isolates were recovered from different sources and fully characterized. The antimicrobial susceptibility test was performed for *K. pneumoniae* isolates against 12 different antibiotics. The results indicate that all collected *K. pneumoniae* are MDR except two isolates (KP13 & KP20). Additionally, the majority (39/40) of *K. pneumoniae* isolates have a m-MAR index above 0.2, which reached a level above 0.5 and > 0.9 in 80% (32/40) and 22.5% (9/40) of *K. pneumoniae* isolates, respectively (Fig. 1). *K. pneumoniae* isolates exhibited a resistance rate to antibiotics ranges from 30 to 87.5%. *K. pneumoniae* isolates were resistant to the last choice antibiotics as meropenem (47.5%), and tigecycline (57.5%) (Supplementary Figure S1).

### Phage isolation and host range

A lytic phage targeting *K. pneumoniae* was isolated from sewage using *K. pneumoniae* isolate KP17 (strain number CCASU-2024-75) as the host bacteria. The isolated phage was named as vB_KpnP_KP17 according to the ICTV guidelines. Considering the lytic potential of isolated phage, vB_KpnP_KP17 exhibited a narrow host range and lysed only 7 out of forty *K. pneumoniae* isolates. Of note that, these lysed bacteria had a wide range of m-MAR indices ranging from 0.25 to 0.67. The phage host range was analyzed by spotting 10 µL of isolated phage on a bacterial lawn of *K. pneumonia*e isolates and other bacterial species. The isolated phage vB_KpnP_kP17 exhibited a lytic activity against 17.5% (7/40) of *K. pneumoniae* isolates, but no lytic action on other bacterial species including *E. coli*, *P. aeruginosa*, *S. aureus*, *S. marcescens*, *S.* Typhimurium and *B. cereus*.

### Efficiency of plating (EOP)

The isolated phage vB_KpnP_kP17 was highly productive on 2 *K. pneumoniae* isolates (KP12 and KP35) with EOP values of 0.67 and 0.84, respectively. However, the isolated phage vB_KpnP_KP17 was less productive on one isolate (KP24) with EOP value of 0.07, and inefficient on other sensitive isolates (KP11 and KP32) with EOP values less than 0.001. Importantly, the reference strain *K. pneumoniae* ATCC700603 designed as KP40 was sensitive to the isolated phage vB_KpnP_KP17 using the spot assay. The lytic potential of vB_KpnP_KP17 against *K. pneumoniae* ATCC700603 was further confirmed using the double-overlay plaque method. *K. pneumoniae* ATCC700603 exhibited an inefficient production lower than that of the host strain KP17 upon infection with vB_KpnP_KP17 with EOP value less than 0.001.

### Morphological characterization of vB_KpnP_KP17 using TEM

**Fig. 2:**
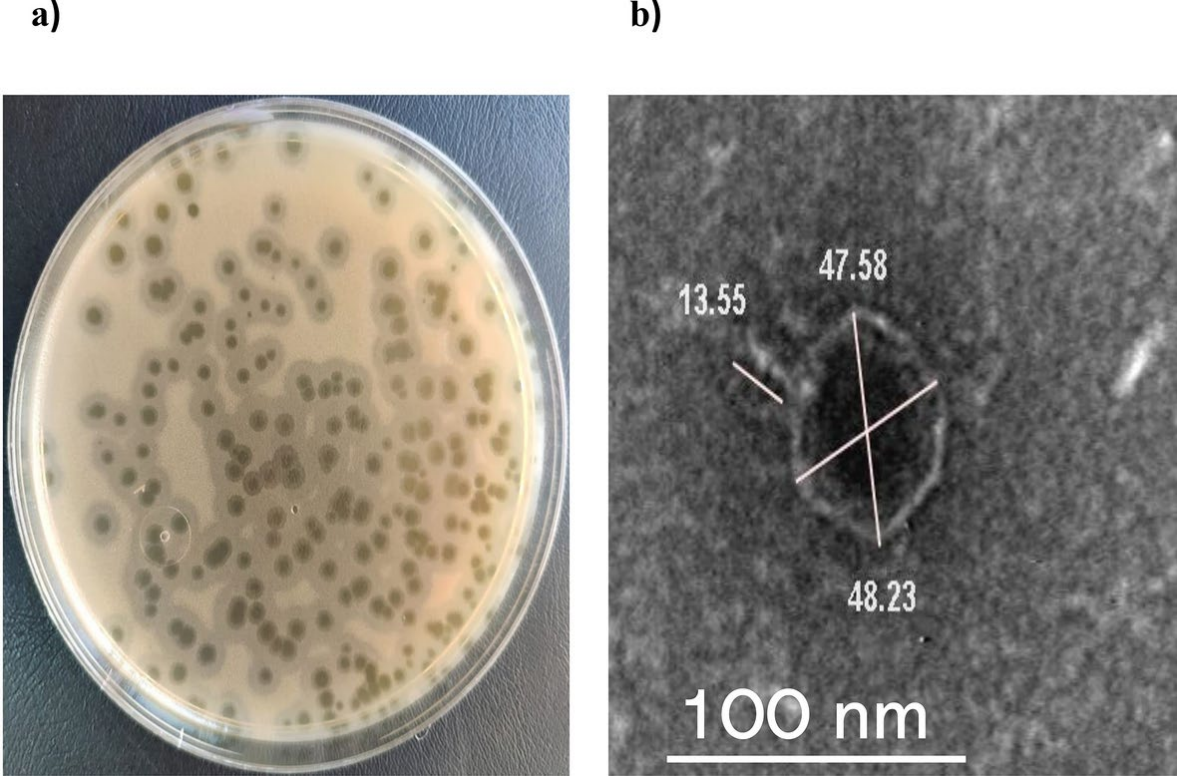
Isolation and characterization of vB_KpnP_kP17. **a** Plaque overlay assay. Isolated phage vB_KpnP_KP17 produced clear plaques with translucent halos after overnight culture with its host KP17. **b** Transmission electron micrograph. The isolated phage vB_KpnP_KP17 was negatively stained with 2% (w/v) phosphotungstic acid, and the image was obtained under transmission electron microscopy at a scale bar of 100 nm

The plaque assay indicates that vB_KpnP_kP17 had a unique plaque shape on host bacteria (KP17) (Fig. 2a) that showed a transparent circle (2 mm) surrounded by a double halo zone (6 mm) in diameter. The shape of plaques on plates is a phenotypic indicator of depolymerase enzyme activity. Negative staining by TEM (Fig. 2b) indicates a phage with an icosahedral head of approximately 48 nm in diameter and a very short tail of 13.5 nm in length.

### Physicochemical stability of vB_KpnP_KP17

**Fig. 3:**
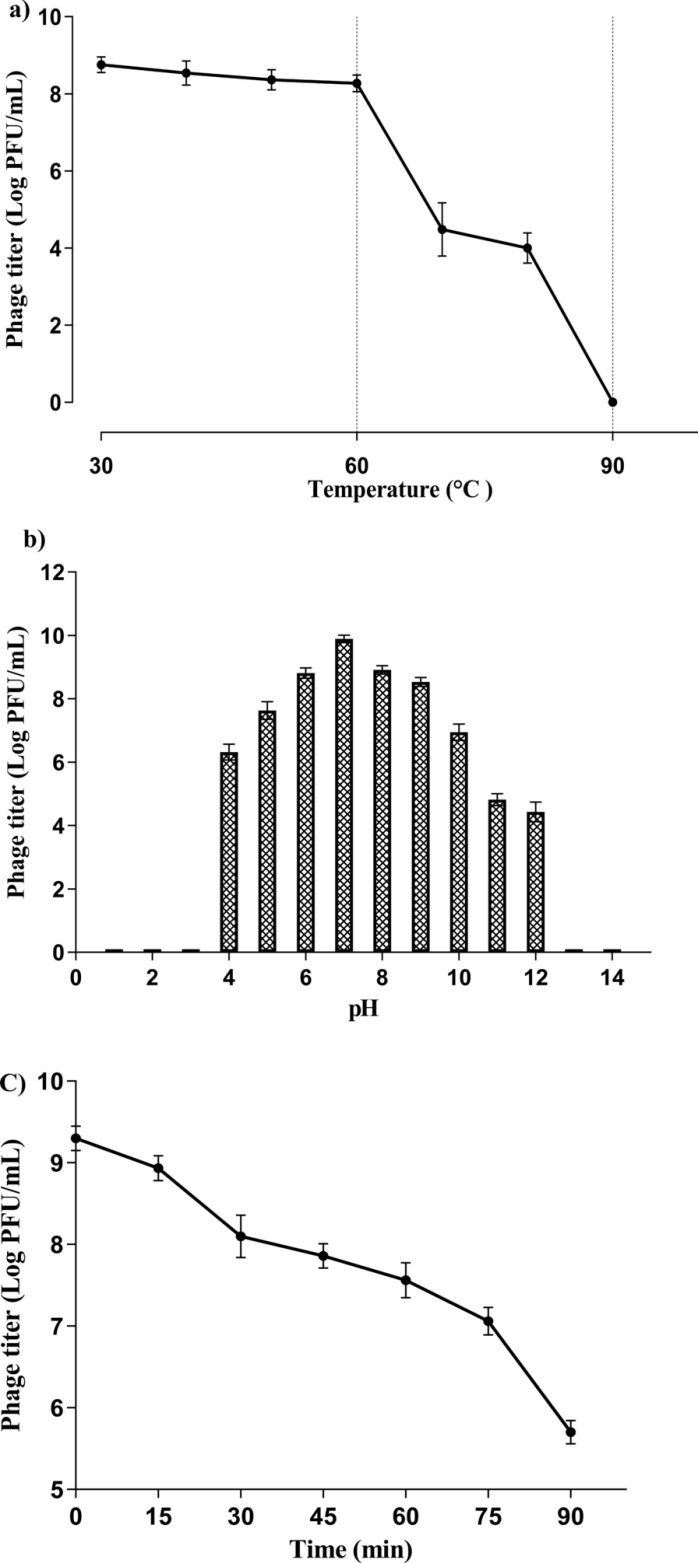
Determination of environmental stability of VB_KpnP_KP17. **a** Temperature stability; **b** pH stability; and **c** UV stability

The isolated phage vB_KpnP_kP17 was thermally stable over a range of 30–60 °C. Afterwards, the phage titer decreased suddenly by about 4.5 log at 70 and 80 °C, and the phage infectivity was completely lost at 90 °C (Fig. 3a). Similarly, the phage particles were challenged against different pH for 1 h. Maximum activity of vB_KpnP_KP17 was observed at pH 7, and the phage maintained a stable titer within a pH range of 6–9. At acidic conditions, there was a gradual decrease in phage titer, and the phage particles were absent at pH 3. The phage population showed a complete loss of activity in extremely alkaline conditions (pH > 12) (Fig. 3b). The stability of vB_KpnP_KP17 in chloroform showed a fixed number of phage particles at all tested concentrations of chloroform. The phage vB_KpnP_kP17 under UV light (Fig. 3c) exhibited a gradual decrease in titer overtime, and its titer reduced by about 1.8 log after 60 min of UV light exposure. The titer of vB_KpnP_KP17 decreased by about 3.6 log following UV exposure for 90 min.

### One-step growth curve and time killing assay

**Fig. 4:**
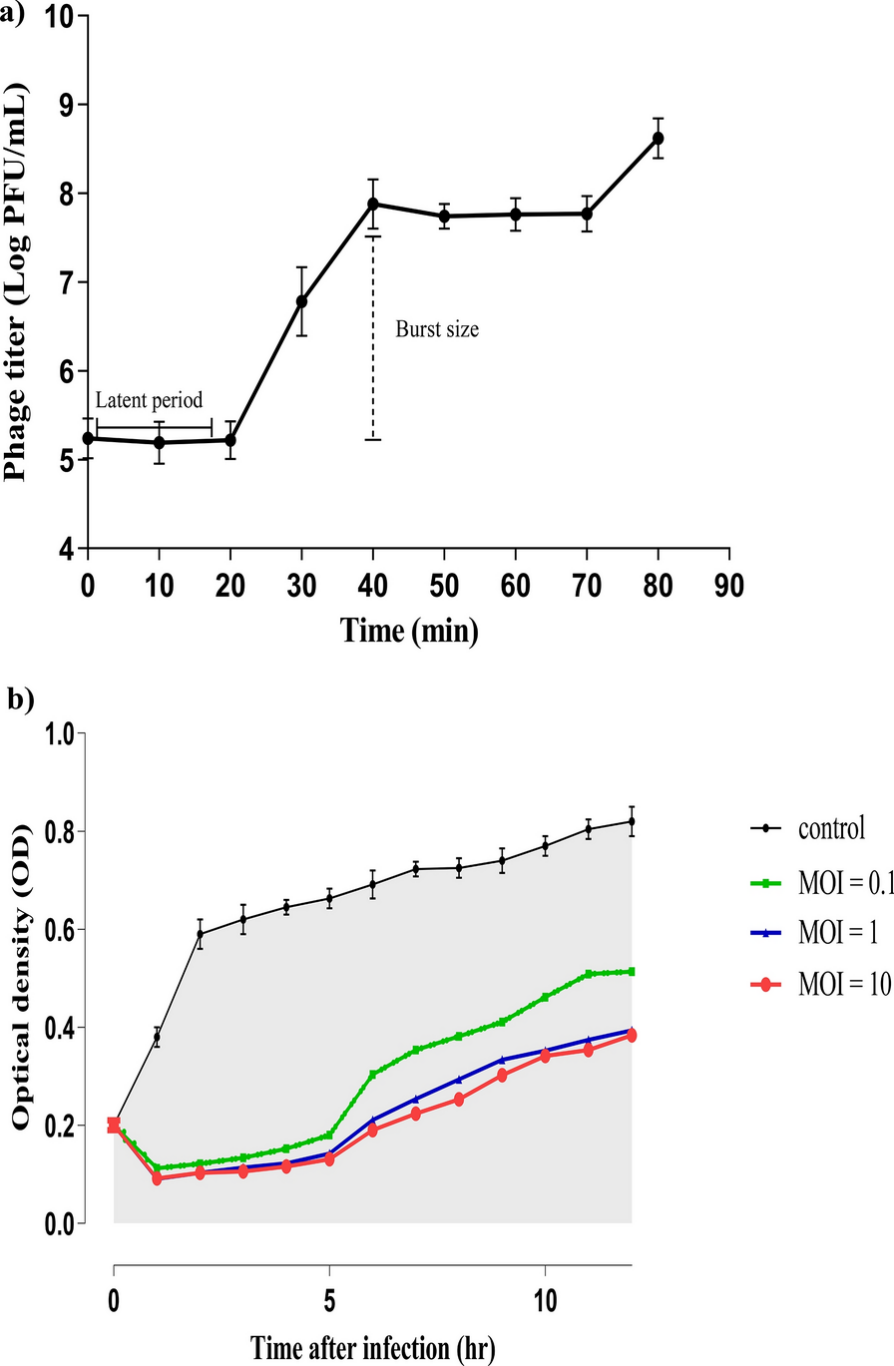
Lytic activity of vB_KpnP_KP17. **a** One-step growth curve. The graph illustrates the initial number of invading virions and progenies released after lysis of host KP17. The phage latent period was 20 min and the phage burst size was 331 PFU/mL. **b** In vitro bacteriolytic activity. The isolated phage vB_KpnP_KP17 and indicator host KP17 were incubated at various MOIs (0.1, 1 and 10) for 12 h. The change in bacterial turbidity was recorded spectrophotometrically at 570 nm in a microtiter plate each 1 h for 12 h

The lytic cycle of isolated phage vB_KpnP_KP17 was summarized in the total number of released progenies per infected cell and the adsorption speed. The latent period was determined and found to be 20 min and the phage burst size was calculated and found to be 331 PFU/mL (Fig. 4a). The phage vB_KpnP_kP17 achieved its peak point of titer only after 40 min, and the phage titer was stabilized within an interval between 40 and 70 min. As shown in Fig. 4b, the bacteriolytic activity of vB_KpnP_kP17 on host bacteria (KP17) was tested at various MOIs for 12 h. The growth of planktonic cells was retarded by isolated phage at all tested MOIs. The phage-treated cultures were slightly clear within the first 5 h. Afterwards, the gradual increase in turbidity was observed within the interval between 6 and 12 h.

### Biofilm inhibition assay

**Fig. 5:**
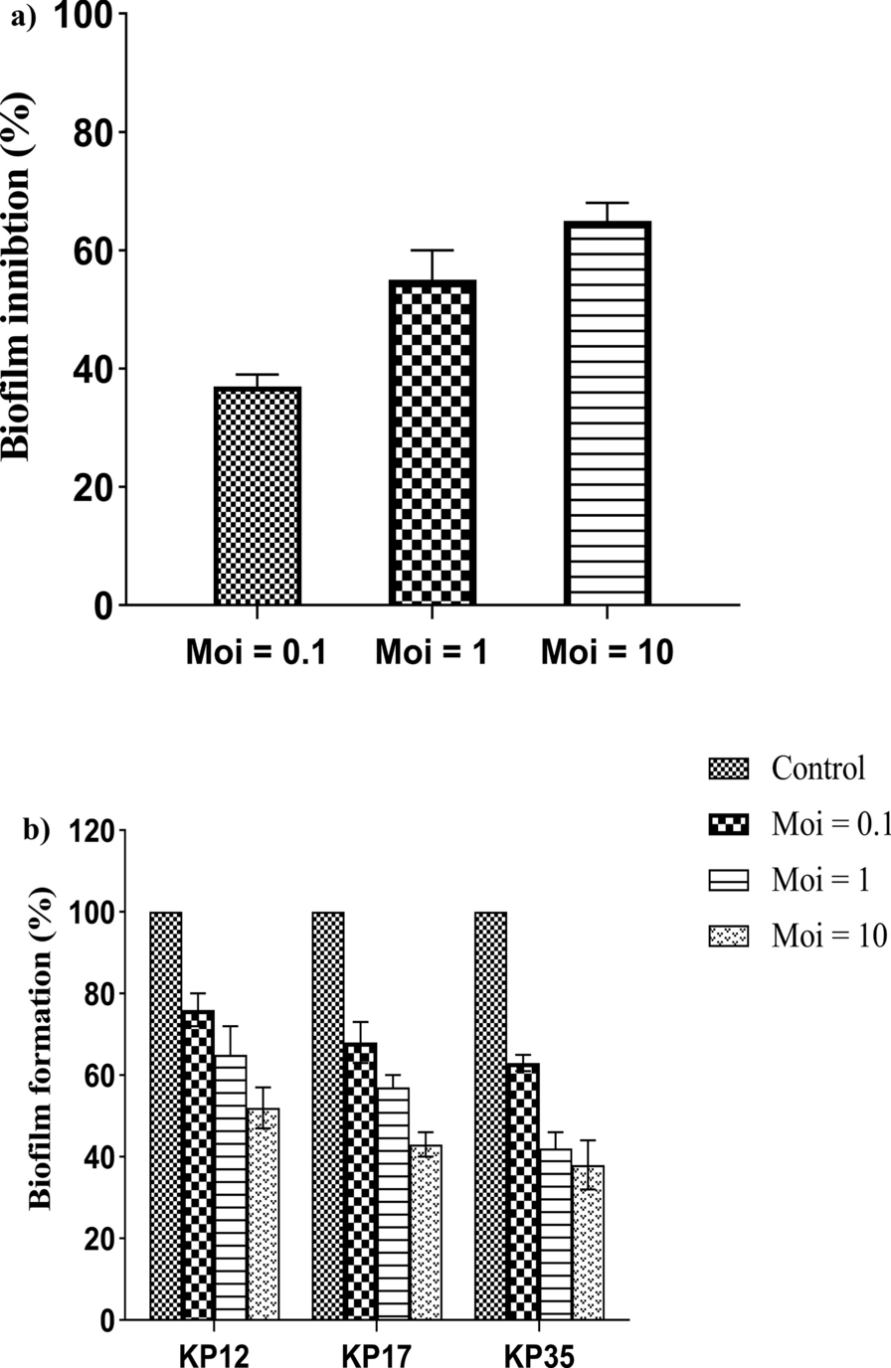
Antibiofilm activity of vB_KpnP_kP17 was detected by the crystal violet technique. **a** Percent of biofilm inhibition by vB_KpnP_KP17 at different MOIs (0.1, 1 and 10) on host bacteria KP17. **b** Percent of biofilm degradation by vB_KpnP_KP17 on tested *K. pneumonia* isolates KP12, KP17 and KP35

Biofilm assay was determined by the crystal violet staining technique. The capacity of biofilm formation among *K. pneumoniae* isolates was different in strength, where 4 (10%) of isolates were strong, 17 (42.5%) of isolates were moderate, and 19 (47.5%) of strains were weak. Regarding biofilm inhibition, the isolated phage vB_KpnP_kP17 and the host bacteria (KP17) were co-cultured simultaneously at different MOIs in a microtiter plate. The crystal violet assay revealed that vB_KpnP_KP17 significantly inhibited the biofilm mass of host bacteria (KP17) as shown in Fig. 5a. Considering the biofilm degradation, the preformed biofilms of *K. pneumoniae* isolates (KP12, KP17 and KP35) were significantly removed in a dose-dependent manner, where the isolated phage vB_KpnP_KP17 at MOI of 10 degraded the biofilm of tested bacteria better than MOIs of 0.1 and 1 (Fig. 5b).

### Whole genome sequencing

**Fig. 6:**
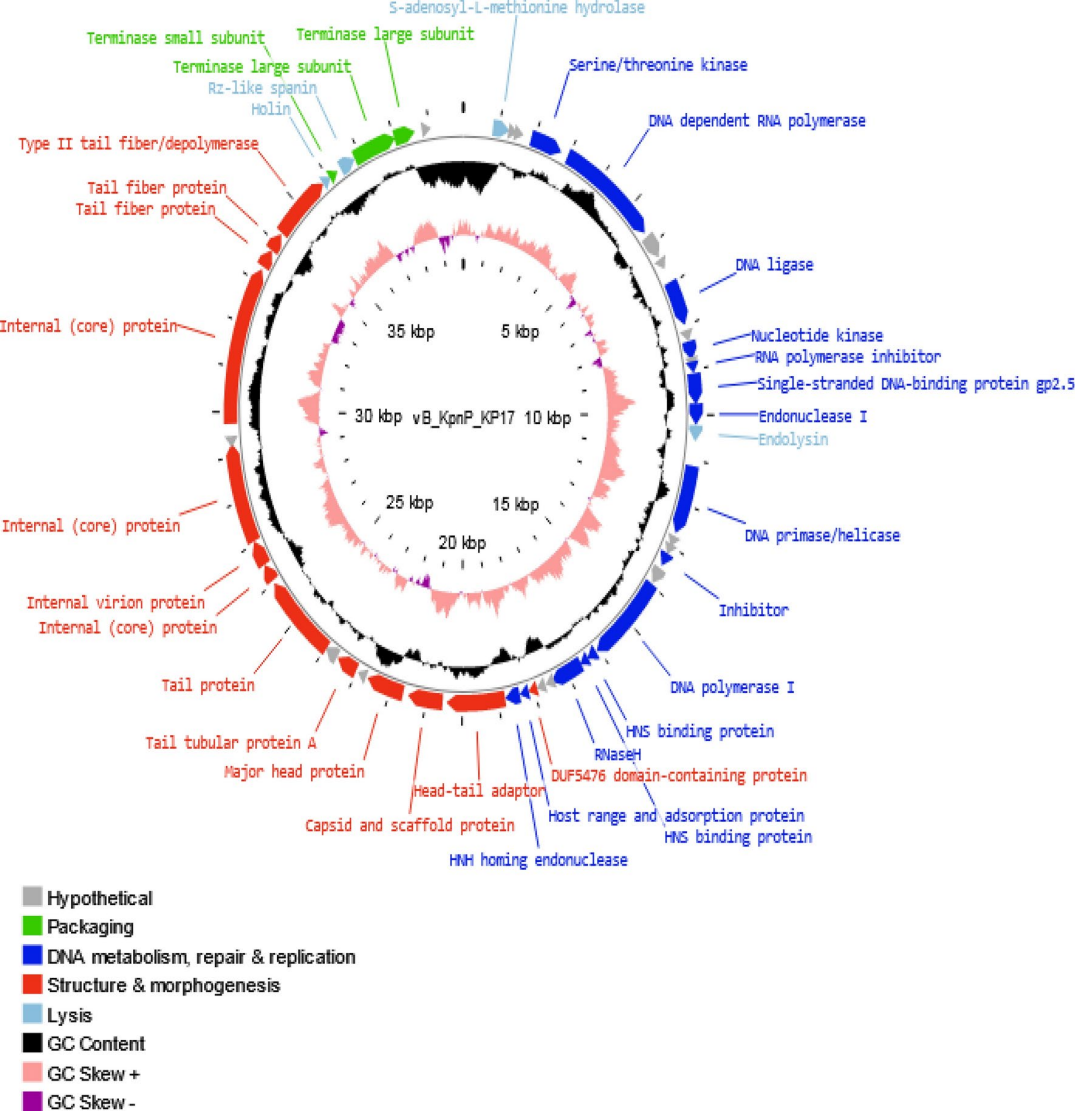
Genomic circular map of vB_KpnP_kP17. The open reading frames (ORFs) are represented in different colors according to associated functions: hypothetical (grey); DNA packaging (green); DNA metabolism, replication and repair (blue); structure (red); lysis (light blue). The inner most circle represents the GC skew and the middle circle represents the GC content

The genome of isolated phage vB_KpnP_kP17 is a linear dsDNA with a length of 39,936 bp and GC content of 52.85%. The annotated genome is composed of 50 open reading frames (ORFs), and these genes are transcribed in a forward direction except one gene (ORF 40). Among these 50 genes, 35 coding sequences were functionally annotated, while the other genes were labeled as hypothetical proteins (Fig. 6). The InterPro tool revealed no predicted function for the hypothetical proteins. The predicted proteins range from 36 to 1185 amino acid residues in length. The genome of vB_KpnP_kP17 encompasses all core genes. The annotated ORFs were classified according to modular function into 4 groups including virion morphogenesis (13 ORFs: 7 ORFs for capsid structure and 6 ORFs for tail structure), DNA metabolism, repair and replication (15 ORFs), DNA packaging (3 ORFs), and four ORFs express enzymes for cell lysis (Table S1). None of the screened ORFs encodes proteins that threaten the applicability and safety of isolated phage, such as antibiotic resistance, bacterial virulence, and toxicity genes. Moreover, the lysogenic-related markers such as integrases and transposases are absent in the annotated phage genome and therefore, a virulent life cycle was adopted by vB_KpnP_kP17.

### Phylogenetic analysis

**Fig. 7:**
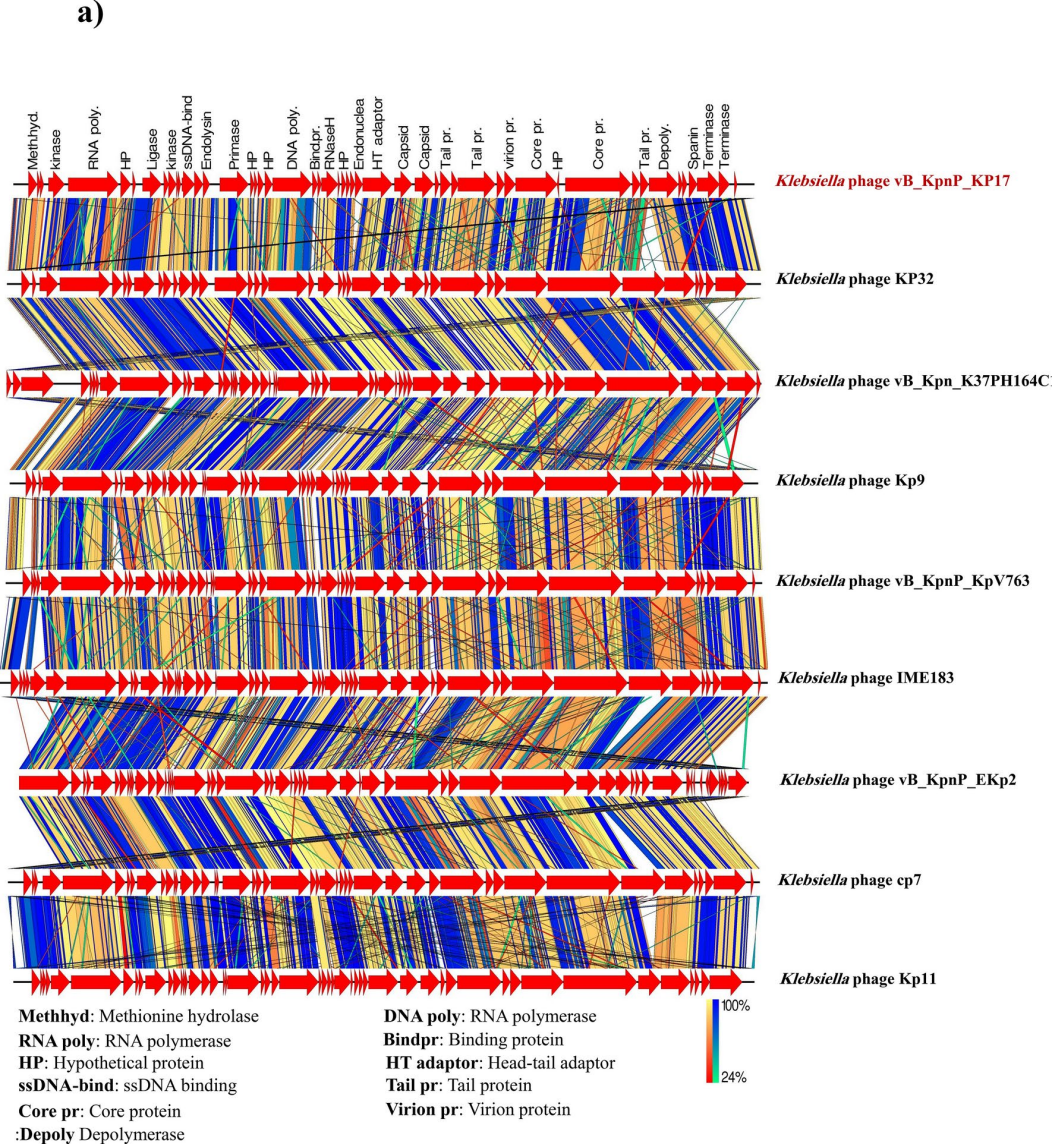

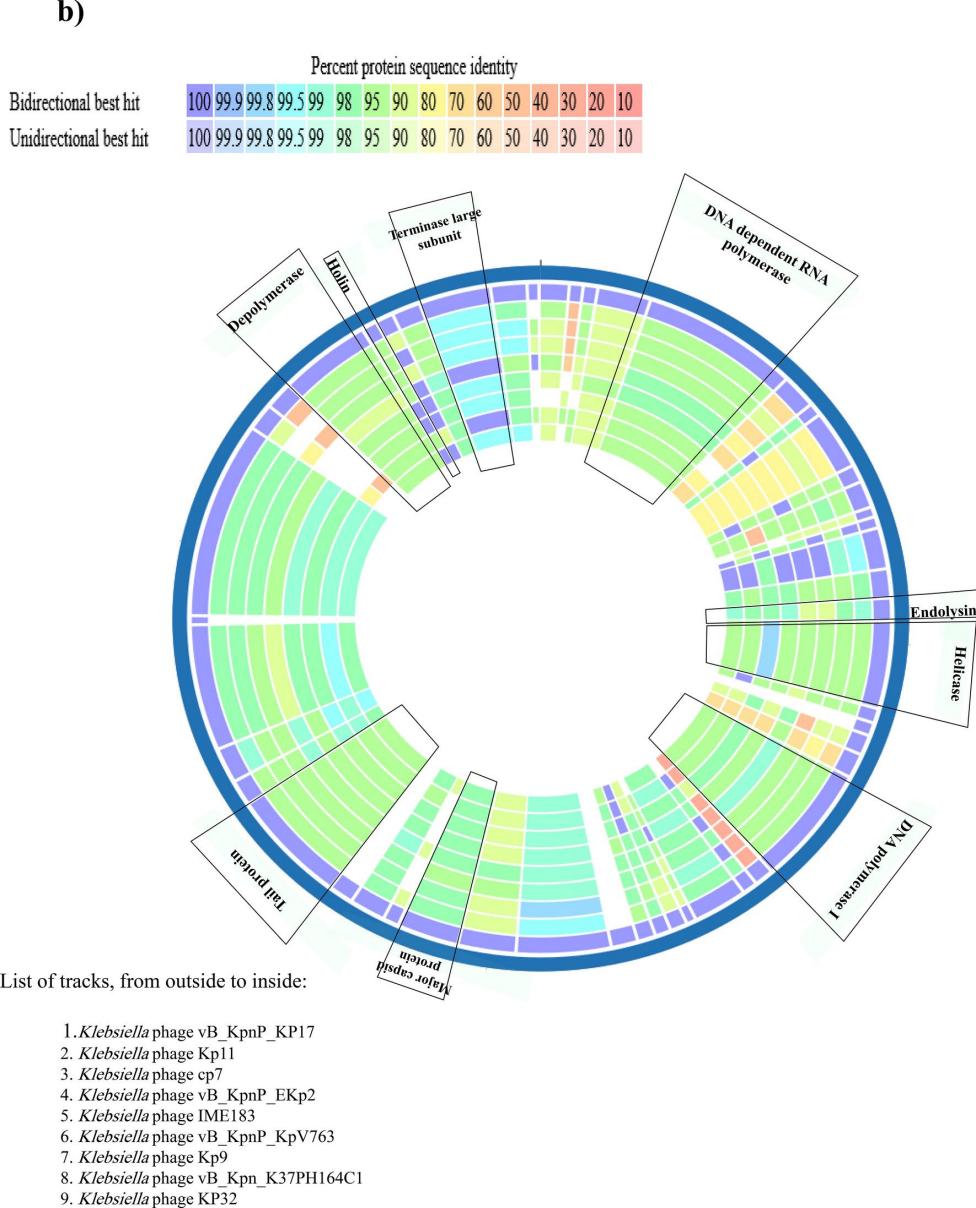
Comparative genomic analysis of vB_KpnP_KP17 and homologous phages. **a** Pairwise comparisons of coding sequence similarities were performed by the Easyfig program, and these gene similarities are displayed by percent identity. Red arrows indicate open reading frames and shaded blue bands represent homology between nucleotide sequences. **b** Proteomic comparison based on the bidirectional BLASTp hits is represented as a circular diagram

**Fig. 8:**
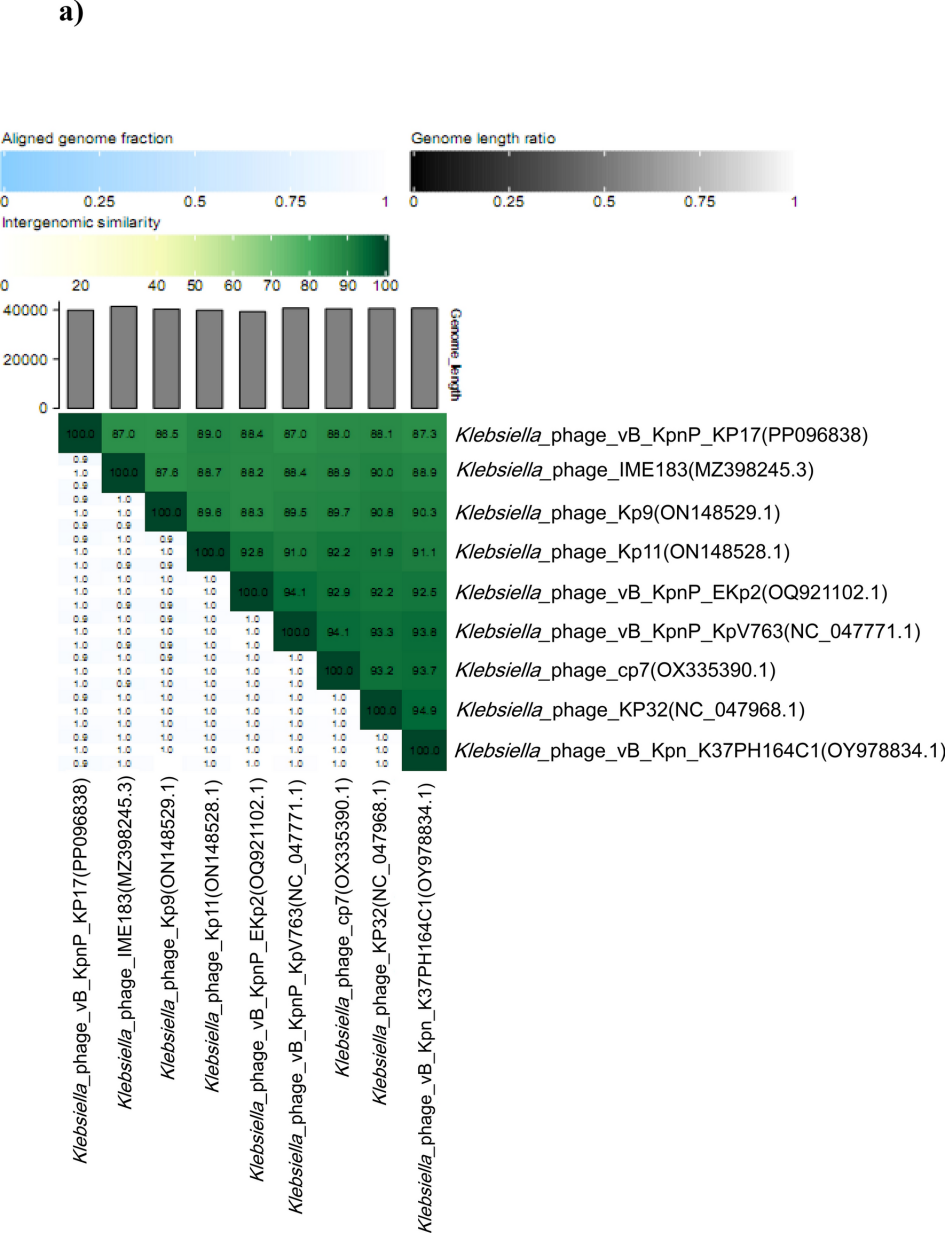

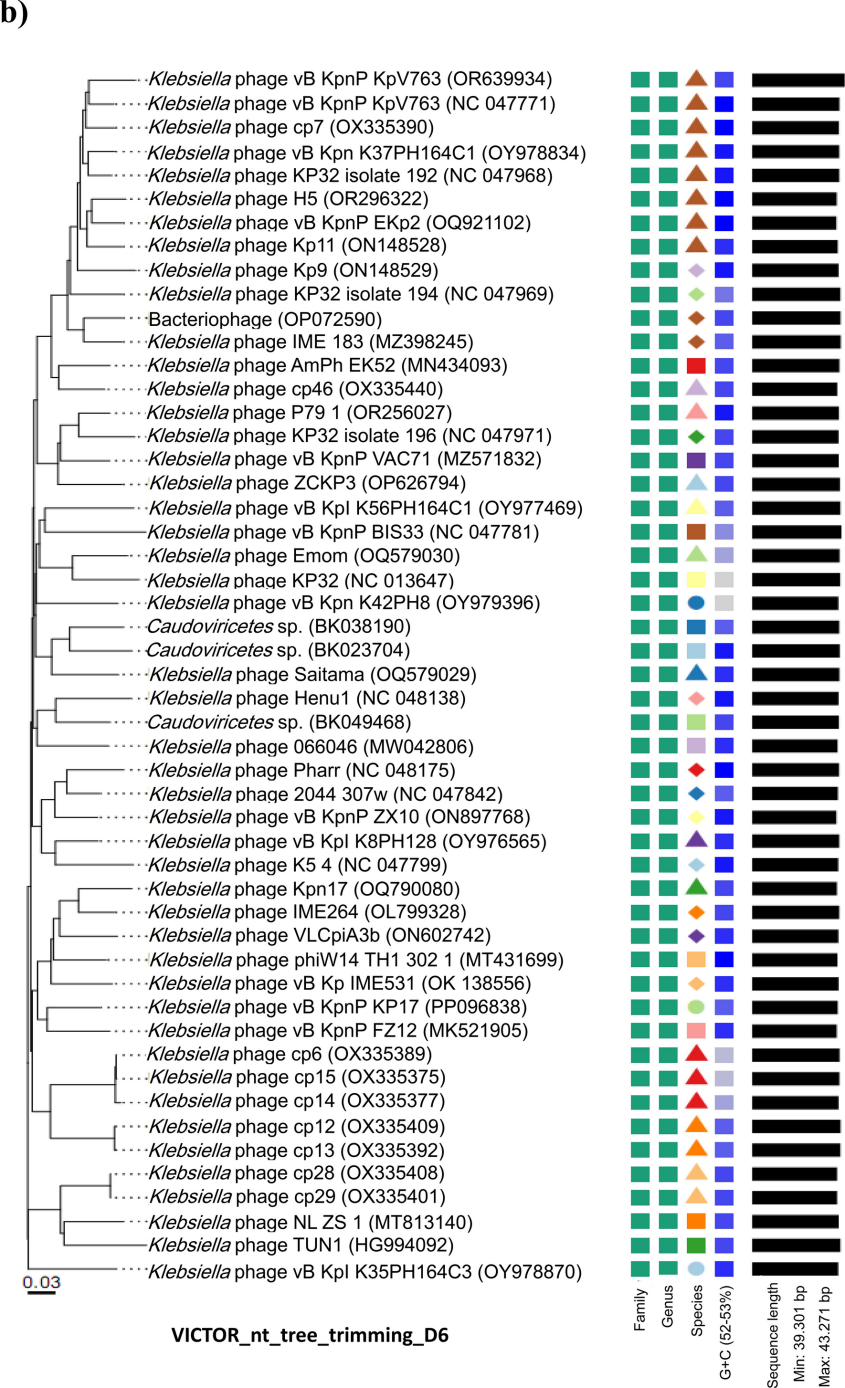
Phylogeny of the isolated phage vB_KpnP_KP17. **a** VIRDIC heat map was created to compare vB_KpnP_kP17 and closely related phages based on BLASTn top hits. **b** Genome-BLAST distance phylogenetic tree was generated by VICTOR using the complete genome sequence of vB_KpnP_kP17 and members of the *Autographiviridae* family

**Fig. 9:**
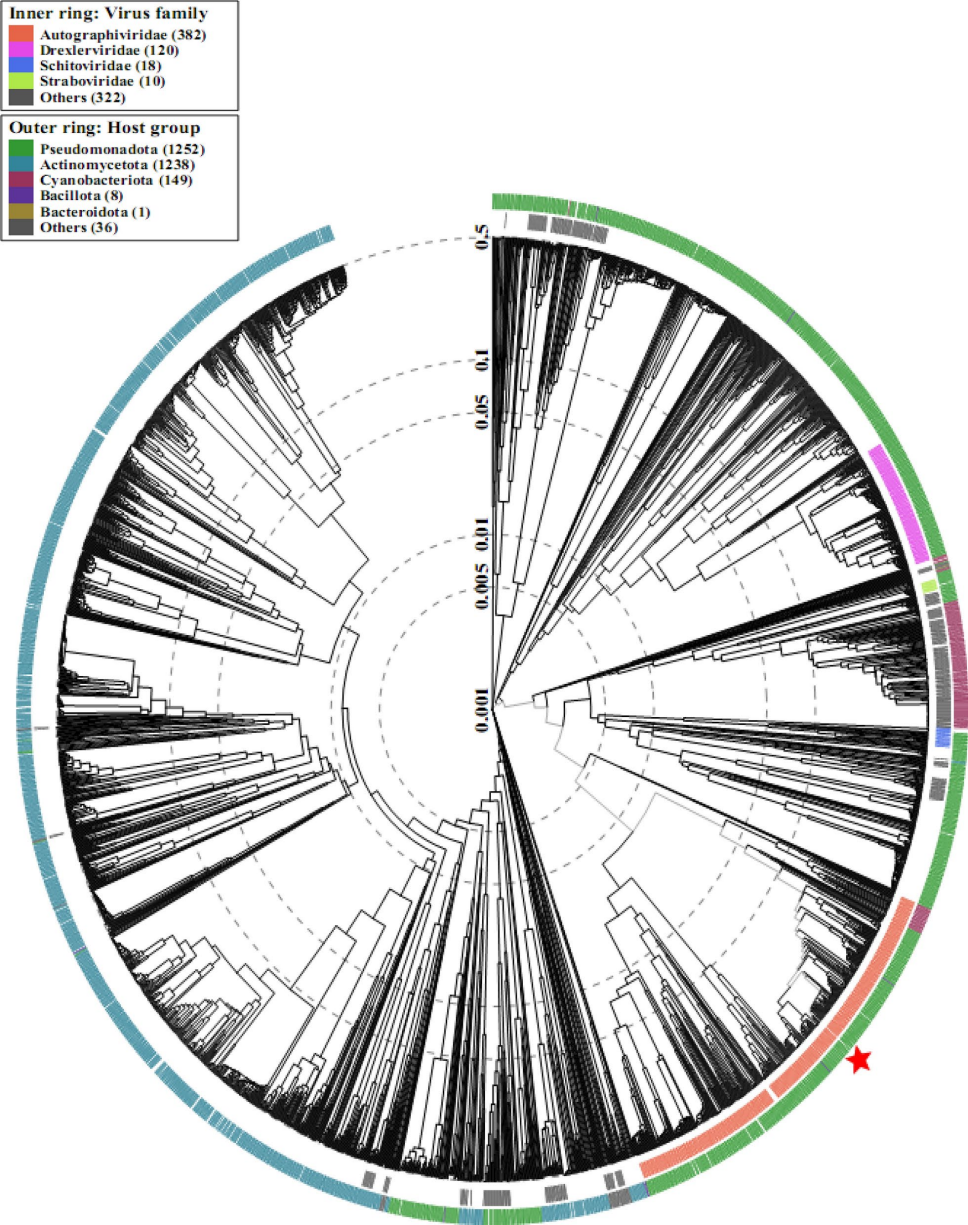
Viral proteomic tree based on genome-wide similarities. ViPTree of vB_KpnP_KP17 and other *Klebsiella* phages was represented as a circle. Taxonomic information of viral and host families is represented as colored inner and outer rings, respectively while the red star represents vB_KpnP_KP17

**Fig. 10:**
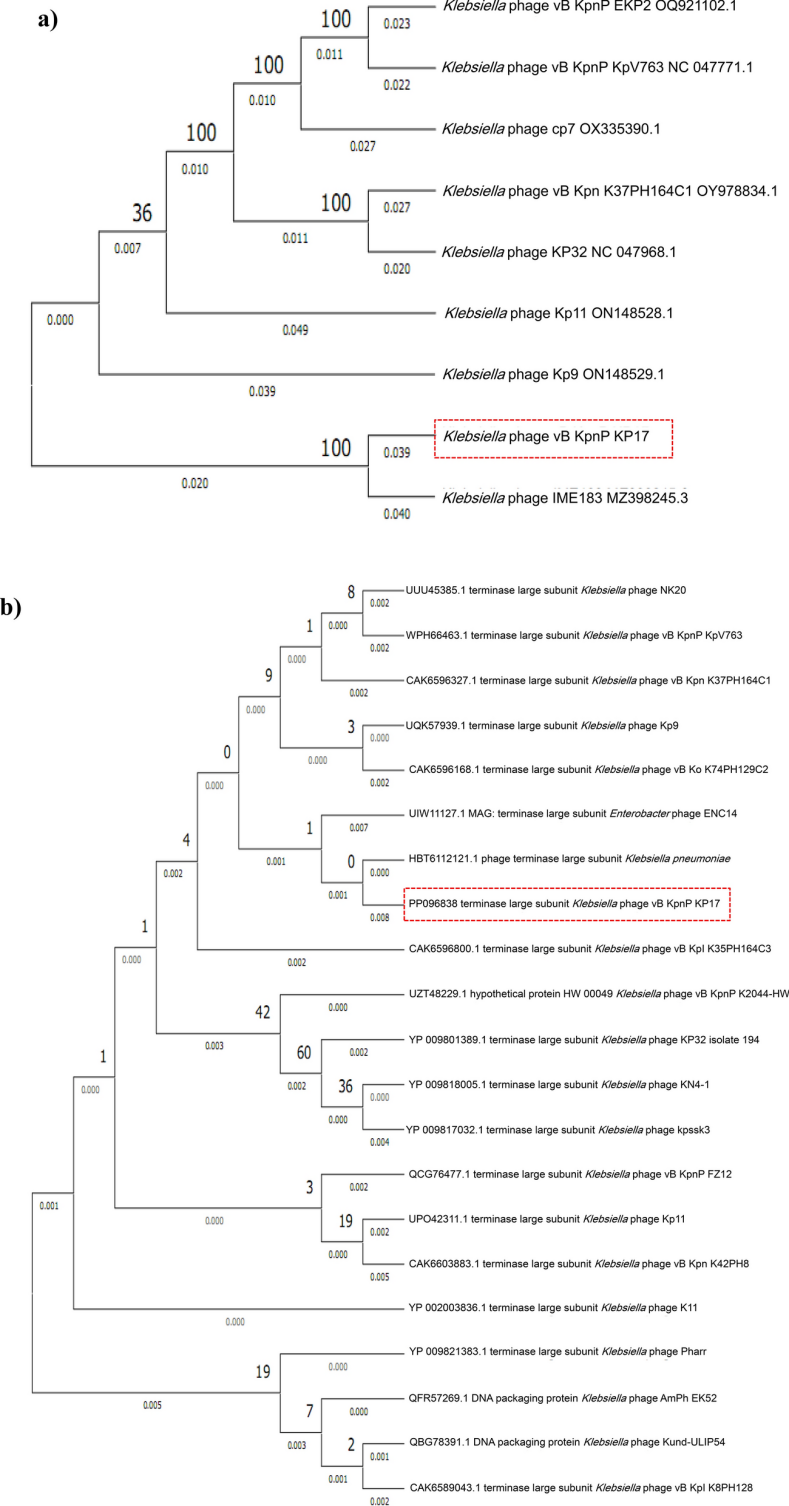

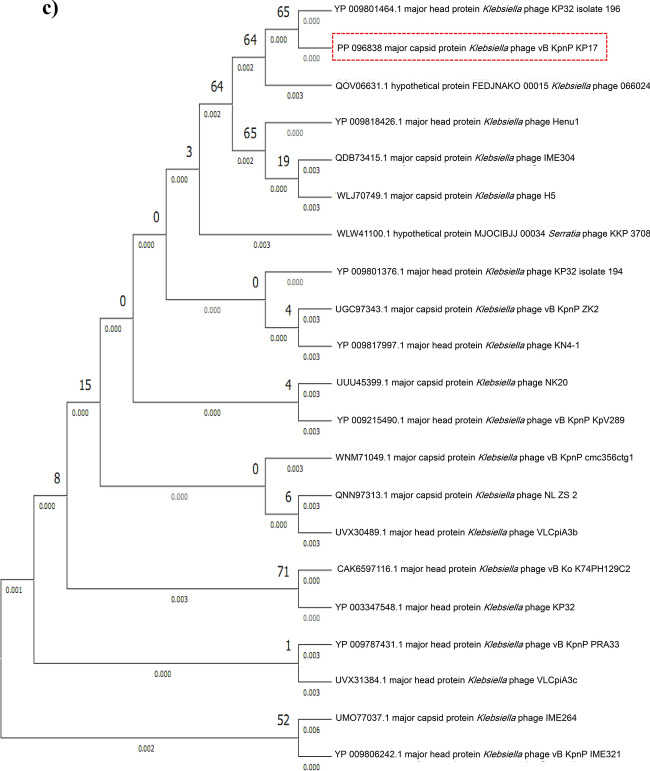

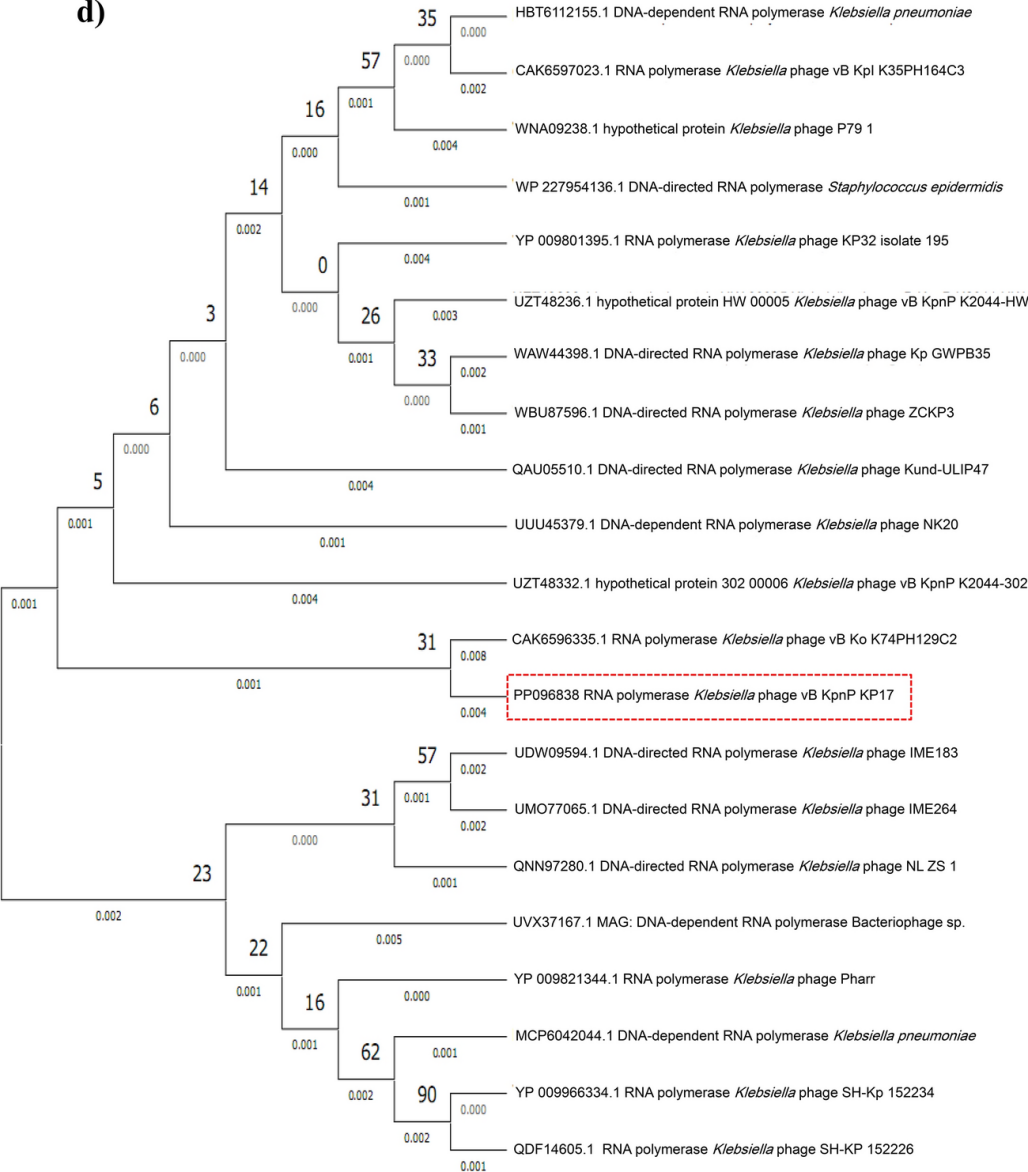
Maximum likelihood trees based on amino acid sequences of vB_KpnP_KP17 and other *Klebsiella* phages. **a** Complete genome; **b** Terminase large subunit; **c** Major capsid protein; **d** RNA polymerase. The phylogeny trees were constructed by MEGA software using the neighbor-joining method

The comparative sequence alignment of vB_KpnP_KP17 with the genomes of similar phages in the NCBI database showed that isolated phage shared the highest nucleotide homology (identity: 94.79%) with *Klebsiella* phage KP32 (GenBank: NC_047968.1), followed by *Klebsiella* phage vB_Kpn_K37PH164C1 (GenBank: OY978834.1), *Klebsiella* phage Kp9 (GenBank: ON148529.1), *Klebsiella* phage vB_KpnP_KpV763 (GenBank: NC_047771.1), *Klebsiella* phage IME183 (GenBank: MZ398245.3), *Klebsiella* phage vB_KpnP_EKp2 (GenBank: OQ921102.1), *Klebsiella* phage cp7 (GenBank: OX335390.1), *Klebsiella* phage Kp11 (GenBank:ON148528.1), with percent identity of (94.71, 93.97, 93.88, 93.81, 93.71, 93.3 and 93.28%); respectively (Supplementary Table S2). Genome synteny analysis using MAUVE software was performed to highlight the variable genome segments between vB_KpnP_KP17 and close phages homologs (Supplementary Figure S2). The data revealed that homologous segments were represented by the same color and linked together by lines. All homologous genomes contain four local collinear blocks (LCBs). The isolated phage vB_KpnP_KP17 has a high collinearity with homologous phages at protein-encoding regions, typically genes encoding structural proteins, DNA packaging, DNA metabolism, repair and replication. In addition, vB_KpnP_KP17 genome and its close homologs were compared by Easyfig revealing a high level of synteny (Fig. 7a). The size and arrangement of ORFs in vB_KpnP_KP17 and aligned genomes were compared along with their transcriptional orientation. Moreover, the amino acid sequence of isolated phage vB_KpnP_KP17 was compared with its related best hits using BLASTp analysis via PATRIC software (Fig. 7b). Importantly, vB_KpnP_KP17 and its related phages share substantial homology based on amino acid sequence identity above 95% for core proteins including RNA polymerase (ORF 5), helicase (ORF 16), DNA polymerase (ORF 21), major capsid protein (ORF 32), tail protein (ORF 36), holin (ORF 45) and terminase large subunit (ORF 48). The pairwise similarity of related phages was further analyzed by VIRDIC based on aligned genome fraction and genome length ratio (Fig. 8a). VIRDIC heatmap revealed that the isolated phage vB_KpnP_KP17 shared the highest intergenomic similarity with percent identity of 89% with *Klebsiella* phage Kp11, followed by *Klebsiella* phage Kp9, *Klebsiella* phage vB_KpnP_EKp2, *Klebsiella* phage KP32, *Klebsiella* phage cp7, *Klebsiella* phage vB_Kpn_K37PH164C1, and both *Klebsiella* phage IME183, *Klebsiella* phage vB_Kpnp_KpV763 with percent intergenomic similarity of 88.5, 88.4, 88.1, 88, 87.3, 87%, respectively. Furthermore, shared-protein network analysis was performed using vConTACT (Supplementary Fig. S3). This analysis identified that vB_KpnP_KP17 shared a viral cluster (VC) with 59 phages of *Autographiviridae* family. To further refine the taxonomy, the VICTOR analysis showed that vB_KpnP_KP17 and other 50 phages including close homologs were grouped as 1 cluster at the family level, 1 cluster at the genus level and 39 clusters at the species level (Fig. 8b). The generated analyses using vConTACT and VICTOR revealed that vB_KpnP_KP17 was most closely related to *Klebsiella* phage KP32 (GenBank Acc.No. NC_047968.1). Additionally, a global protein-based phylogenetic tree was constructed using ViPTree to compare vB_KpnP_KP17 proteome with other 852 dsDNA phage proteomes (Fig. 9). More specifically, a circular proteomic tree clustered vB_KpnP_KP17 with other *Klebsiella* phages within the family *Autographiviridae* and the host group *Pseudomonodota* (Supplementary Figure S4). The phylogenetic tree constructed based on whole genome sequence revealed that vB_KpnP_KP17 is cladded together with its close homologs (Fig. 10a). Moreover, the phylogenetic analysis based on major capsid protein, terminase large subunit and RNA polymerase was further performed indicating a higher relatedness of these proteins to the previously characterized phages proteins (Fig. 10b–d).

## Discussion

Multidrug resistance (MDR) by pathogens causes a public health threat owing to antibiotics misuse. Bacterial antimicrobial resistance was responsible for more than 1.2 million global deaths in 2019, with a predicted killing record of 10 million deaths annually by 2050 unless urgent global action is taken (KWK Tang et al. 2023a, b). *K. pneumoniae* is considered one of the most important opportunistic pathogens worldwide owing to its broad resistance profile and multiorgan damage (Sadeqi et al. 2023).

In the present study, the antimicrobial sensitivity testing was performed on 40 K*. pneumoniae* clinical isolates revealing that 95% of tested isolates were MDR. The multiple antibiotic resistance (MAR) index is an efficient parameter to determine the microbial threats to public health. The mean modified-MAR index for these isolates was approximately 0.7, and it was above 0.5 in 80% of tested bacterial isolates. Therefore, the present research was conducted on phage therapy as a promising alternative to antibiotics against MDR *K. pneumoniae.* Virulent phages offer many advantages over antibiotics as being bactericidal with exponential increase in virion numbers, namely "auto dosing" and ideal for personalized therapy without side effects on normal flora (Loc-Carrillo and Abedon 2011).

A phage vB_KpnP_KP17 was isolated herein, and its lytic ability against *K. pneumoniae* was phenotypically detected by a clear zone on a bacterial lawn. The isolated phage has a relatively narrow host range and was lytic against 7 out of 40 K*. pneumoniae* isolates. However, all sensitive *K. pneumoniae* isolates to vB_KpnP_KP17 were MDR and exhibited a m-MAR index ranges from 0.25 to 0.67 (mean = 0.5). Moreover, the EOP values were variable among phage-sensitive isolates, which could be due to the difference in relative binding affinity between the phage tail spike and bacterial surface molecules (Han et al. 2024). Of note that, narrow host range is a common feature among *Klebsiella* phages due to variations and manipulations in bacterial capsule receptors (Beamud et al. 2023; Wang et al. 2024a, b). The application of a single phage with narrow tropism is a major concern in phage therapy. To tackle this challenge, formulation of isolated phage in a cocktail with other phages targeting different capsular types is recommended to overcome this narrow tropism (Sattar et al. 2023). The major advantages of phage cocktails include a broadened host range, delaying the emergence of mutant variants. An alternative methodology involves a combination of phage with bactericidal antibiotics that improve the lytic efficiency of phage (Chen et al. 2024). Moreover, the isolated phage could be genetically engineered to create a manipulated phage that targets a wide spectrum of *K. pneumoniae* strains (Lenneman et al. 2021).

The morphological characteristics of vB_KpnP_kP17 have been characterized by TEM. The isolated phage vB_KpnP_KP17 is similar to the previously reported *Klebsiella* phages KP34 and TUN1 with icosahedral head and very short tail (Drulis-Kawa et al. 2011; Eckstein et al. 2021). Importantly, the maintenance of phage viability across various environmental stability conditions is the key feature for therapeutic purposes (Islam et al. 2023). For industrial applications, the phage is exposed to common environmental conditions of temperature range of 4–40 °C and pH range of 4–10 during production, storage and treatment (Bai et al. 2022). Interestingly, vB_KpnP_KP17 remains stable at this temperature and pH ranges and is suggested to be successfully formulated. Importantly, resistance of phage at extreme pH levels blocks phage denaturation by irreversible precipitation and coagulation (Malik et al. 2017). Moreover, the bacteriophage that withstands extreme temperatures could maintain its survivability, morphology and efficiency. Therefore, the isolated phage could resist techniques used for the preparation of formulations such as aerosols and possess a good shelf-life (Pradeep et al. 2022). The capacity of vB_KpnP_KP17 to withstand drastic environmental conditions is more suitable for different infection models. For instance, vB_KpnP_KP17 titer remains stable under variable pH conditions, suggesting its therapeutic suitability for *K. pneumoniae* that causes skin, lung and urogenital infections (Martín-Rodríguez et al. 2020). Additionally, the prolonged stability of the isolated phage vB_KpnP_KP17 under UV light suggested that vB_KpnP_KP17 can be administered as a topical product (Fernández et al. 2019).

The one step growth curve indicates that vB_KpnP_kP17 has a latent period of 20 min and a burst size of 331 PFU/cell, which is larger than that of previously isolated *Klebsiella* phages vB_KleS-HSE3 (277 PFU/cell), PG14 (47 PFU/cell), and IME183 (135 PFU/cell), but smaller than vB_Kpn_ZC2 (650 PFU per cell) (Fayez et al. 2023; Mulani et al. 2022; Peng et al. 2020; Xu et al. 2023). The rapid phage adsorption and large release of progenies further confirm the therapeutic efficacy of vB_KpnP_KP17. Phage virulence against *K. pneumoniae* planktonic cells has been determined herein through the time-killing assay. The growth of planktonic cells was greatly inhibited within 5 h of incubation. However, the bacterial turbidity began to rise gradually from 5 h after phage treatment until it reached 12 h. This gradual increase in culture turbidity could be attributed to the development of phage-resistant variants. The primary mechanism is suggested to be the loss of a bacterial capsule due to mutations caused by insertion sequences in glycosyltransferase initiators of capsule production, specifically *wcaJ* and *wbaP* (Hesse et al. 2020). The phage-derived depolymerase enzyme exhibits indirect killing activity by removal of the bacterial capsule (Wang et al. 2024a, b). Owing to its decapsulating action, the depolymerase enzyme attenuates the virulence of *K. pneumoniae*. Therefore, the immune evasion capacity of decapsulated mutants is greatly reduced by the depolymerase enzyme (Chen et al. 2022). Importantly, early studies have revealed that these mutant variants are less virulent and easily removed by the human immune cells (Gu et al. 2012; Z Tang et al. 2023a, b). In this regard, capsule is considered the major virulence determinant that protects *K. pneumoniae* from host immune response, including complement-mediated cell lysis and phagocytosis (Bengoechea and Sa Pessoa 2019). The phage-derived depolymerase enzyme has the capacity to eradicate this protective capsule, leading to restoring the bacterial sensitivity to antibiotics. For instance, the depolymerase enzyme (Dpo71) has the capacity to enhance colistin penetration and accelerate its bactericidal action (Wang et al. 2024a, b). Similarly, the depolymerase enzyme renders *K. pneumoniae* more sensitive to gentamicin at low concentrations, thereby decreasing its toxicity (Bansal et al. 2014). Furthermore, these capsule-free mutants are highly susceptible to phages that utilize a capsule-independent mode of entry (Lourenço et al. 2023). Importantly, a combination of vB_KpnP_KP17 with phages that target decapsulated variants is a promising strategy to eradicate these mutant cells (Knecht et al. 2020; Grazyna Majkowska-Skrobek et al. 2018). Therefore, the depolymerase enzyme derived from vB-KpnP_KP17 could be considered as a promising anti-virulence agent for therapeutic purposes (Kaszowska et al. 2021; Grażyna Majkowska-Skrobek et al. 2016; Wei et al. 2023).

Biofilm formation is a physical defense mechanism against immune cells and antibiotics to exaggerate the chronicity of bacterial infection. The biofilm matrix of bacterial species is generally composed of polysaccharides, DNA, and proteins (Wu et al. 2019). The isolated phage vB_KpnP_kP17 showed a significant capacity to inhibit *Klebsiella* biofilm and degrade the preformed biomatrix in vitro. The antibiofilm activity of vB_KpnP_KP17 could be attributed to depolymerase, which can degrade the polysaccharides. Phage-derived depolymerases have been reported as antibiofilm agents against highly resistant bacteria (P Li et al. 2022; Tian et al. 2021). The results of antibiofilm activity employed by vB_KpnP_KP17 are consistent with *Klebsiella* phages KZag1, vB_KpnS_Kp13 and KP1801 (Horváth et al. 2020; Saqr et al. 2024; Wintachai et al. 2020). Additionally, the biofilms formed by *K. pneumoniae* isolates were significantly degraded in an MOI dependent manner following incubation with vB_KpnP_KP17. Considering its in vitro efficacy, the isolated phage vB_KpnP_KP17 possesses several desirable features for inclusion in a phage cocktail. The isolated phage exhibits strong lytic activity and significant antibiofilm properties. Moreover, vB_KpnP_KP17 could be successfully formulated owing to its stability under various drastic conditions. Importantly, the rapid infectivity with a large burst size further supports the utilization of phage in clinical trials against MDR *K. pneumoniae* infections.

The genome of vB_KpnP_kP17 has been sequenced, and the essential proteins have been predicted by gene annotation. The majority of annotated genes participate in head and tail morphogenesis and DNA metabolism. Furthermore, various tools were employed to identify the closest homologs to vB_KpnP_KP17 and further compare their related genomes. The genomic similarity threshold was set at 70 and 95% to classify the bacteriophage at genus and species levels, respectively (Moraru et al. 2020; Turner et al. 2021). VIRDIC software classified vB_KpnP_KP17 at the same genus level as related homologs, but the intergenomic identity was below the species clustering threshold. The results of VICTOR analysis further confirmed the classification of isolated phage vB_KpnP_KP17 as a new species of the genus *Przondovirus* and *Autographiviridae* family (Liu et al. 2024). The infection cycle of vB_KpnP_kP17 is suggested to start by recognition and adsorption of tail fiber proteins (ORF 42, ORF 43, and ORF 44) to host cell receptors. Additionally, a tail tubular protein (ORF 34) has been linked to domains with enzymatic function to invade the bacterial cells through capsule destruction (North et al. 2019). Generally, a bacteriophage that encodes a high amount of tail spike, tail fiber, base plate, and tail tubular proteins can improve attachment to various binding sites, so broaden the host range (Brzozowska et al. 2017). In the lysis module of vB_KpnP_kP17, a group of lytic proteins such as holin, spanin, and endolysin are produced for host destruction. Holin (ORF 45) makes pores in the cell membrane for endolysin transport. The duration needed for complete host destruction is controlled by the holin enzyme. Spanin (ORF 47) is essential for degradation of inner and outer membranes. Endolysin (ORF 15) has the ability to decompose the peptidoglycan layer of the cell wall to release the mature viral progenies (Pacios et al. 2021). Importantly, hydrolase (ORF 1) is suggested to be a novel enzybiotic owing to its dual function as it forms a small hole in the cell envelope for DNA ejection and destroys organization of the cell wall (Rodríguez-Rubio et al. 2013). In the packaging module, a small subunit of the terminal enzyme (ORF 46) attaches to a large subunit to initiate the packaging process. Terminase large subunits (ORF 48 and ORF 49) deliver the genetic material into the empty head and make some modifications in DNA structure (Lu et al. 2020). Of note that, no tRNA was detected in the isolated phage vB_KpnP_KP17 genome. Importantly, tRNAs are the only translation-related genes in phages to permit the host bacterial ribosomal subunits to decode the genetic information (Rak et al. 2018). Therefore, the absence of tRNA could suggest that the isolated phage vB_KpnP_KP17 is fully dependent on host tRNAs for its purposes to translate genes into proteins (Bailly-Bechet et al. 2007). Importantly, none of the annotated genes encoded proteins of the lysogenic cycle, such as integrases or transposases. The phage genome was screened, and the results confirmed the absence of genes that related to antibiotic resistance, bacterial virulence and toxicity. Based on genome sequencing, the isolated phage vB_KpnP_kP17 is highly anticipated to be safe and applicable as a therapeutic agent to manage *K. pneumoniae* infections (Sharma et al. 2017). Future investigations would be conducted to explore the successful formulation of vB_KpnP_KP17 in a cocktail therapy and in a combination with antibiotics. Additional future research would implement the isolated phage vB_KpnP_KP17 for in vivo studies to address the existing gap knowledge. Further experiments can be taken up to cloning and expression of the annotated proteins with bactericidal activity.

In summary, the current study revealed that vB_KpnP_kP17 is a promising candidate for *K. pneumoniae* infections and biofilm clearance. The physicochemical stability of vB_KpnP_KP17 is critical for flexible formulation of isolated phage in different dosage forms. The findings suggest that the isolated phage vB_KpnP_KP17 is a potential candidate for further in vivo studies to prove its efficacy in the field of phage therapy. Moreover, manipulating vB_KpnP_KP17 to produce its bacteriolytic proteins could be considered for future studies as a direct biocontrol against MDR *K. pneumoniae*. The current study highlights the positive impact of phage therapy in addressing challenges posed by antibiotic resistant strains and persistent infections. Importantly, deep mutational scanning studies in combination with machine-learning techniques could be applied to determine the active sites in phage tails and expand phage spectrum. This strategy would encourage the pharmaceutical industries to produce alternative therapeutics based on phage therapy. One of the main issues about vB_KpnP_KP17 is its narrow host range, which can limit its clinical application. However, some useful strategies could be applied in order to increase the susceptibility of *K. pneumoniae* to vB_KpnP_KP17. For instance, vB_KpnP_KP17 can be included within phage cocktail or in combination with either antibiotics or other phages to achieve a maximum therapeutic efficiency.

## Supplementary Information

Supplementary Material 1 (PPTX 1433 KB)

Supplementary Material 2 (DOCX 40 KB)

Supplementary Material 3 (DOCX 15 KB)

## Data Availability

The data supporting the findings of current study are included in the article and supplementary material.

## Abbreviations

MDR: Multidrug resistant
MAR: Multiple antibiotic resistance
TEM: Transmission electron microscopy
PFU: Plaque forming unit
ORFs: Open reading frames
CDS: Coding sequence
MOI: Multiplicity of infection
EOP: Efficiency of plating
TSA: Tryptone soy agar
PBS: Phosphate buffered saline
CLSI: Clinical and laboratory standards institute
ICTV: International Committee Taxonomy of Viruses
OD: Optical density
CFU: Colony forming unit
BLAST: Basic local alignment search tool
