# Supplementary material for: Analysis of a novel phage as a promising biological agent targeting multidrug resistant Klebsiella pneumoniae
Source: AMB Express. 2025 Mar 5;15:37. doi: 10.1186/s13568-025-01846-0 (PMC11882492; doi:10.1186/s13568-025-01846-0)
Supplement: Supplementary file 1 — Supplementary Material 1 (PPTX 1433 KB) [file 13568_2025_1846_MOESM1_ESM.pptx]

## Slide 1
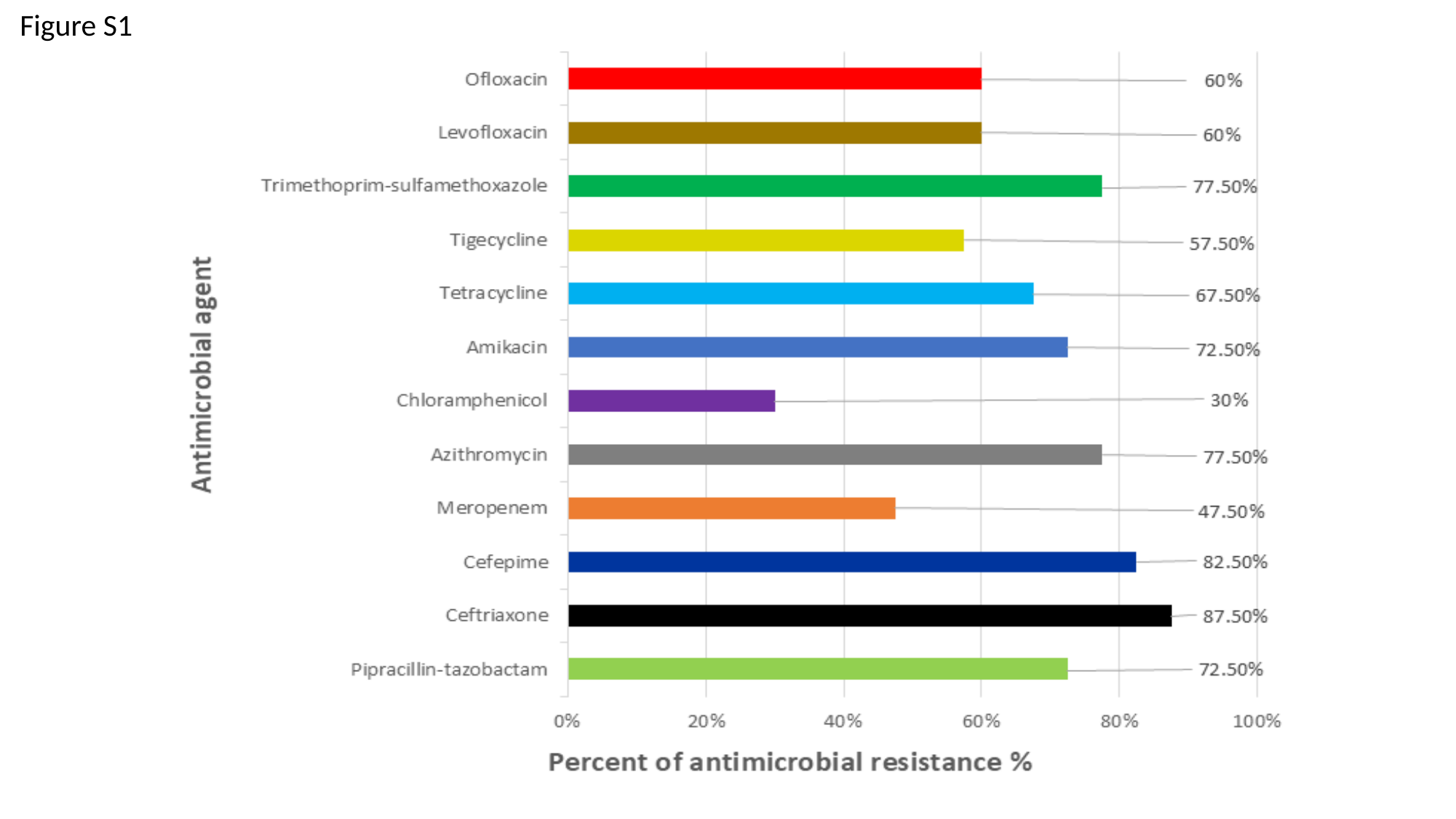

Figure S1

## Slide 2
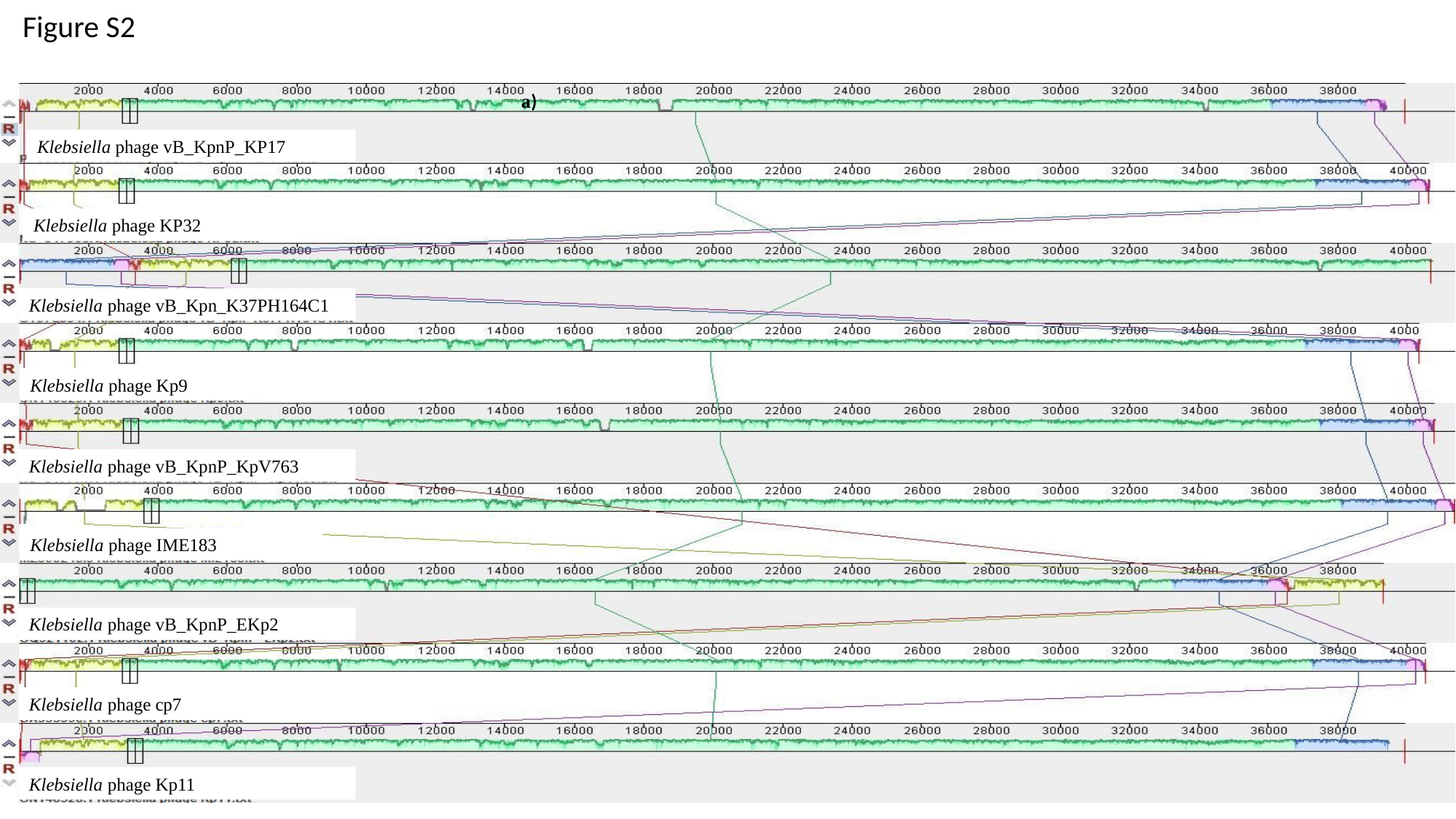

Figure S2
a)
Klebsiella phage vB_KpnP_KP17
Klebsiella phage KP32
Klebsiella phage vB_Kpn_K37PH164C1
Klebsiella phage Kp9
Klebsiella phage vB_KpnP_KpV763
Klebsiella phage IME183
Klebsiella phage vB_KpnP_EKp2
Klebsiella phage cp7
Klebsiella phage Kp11

## Slide 3
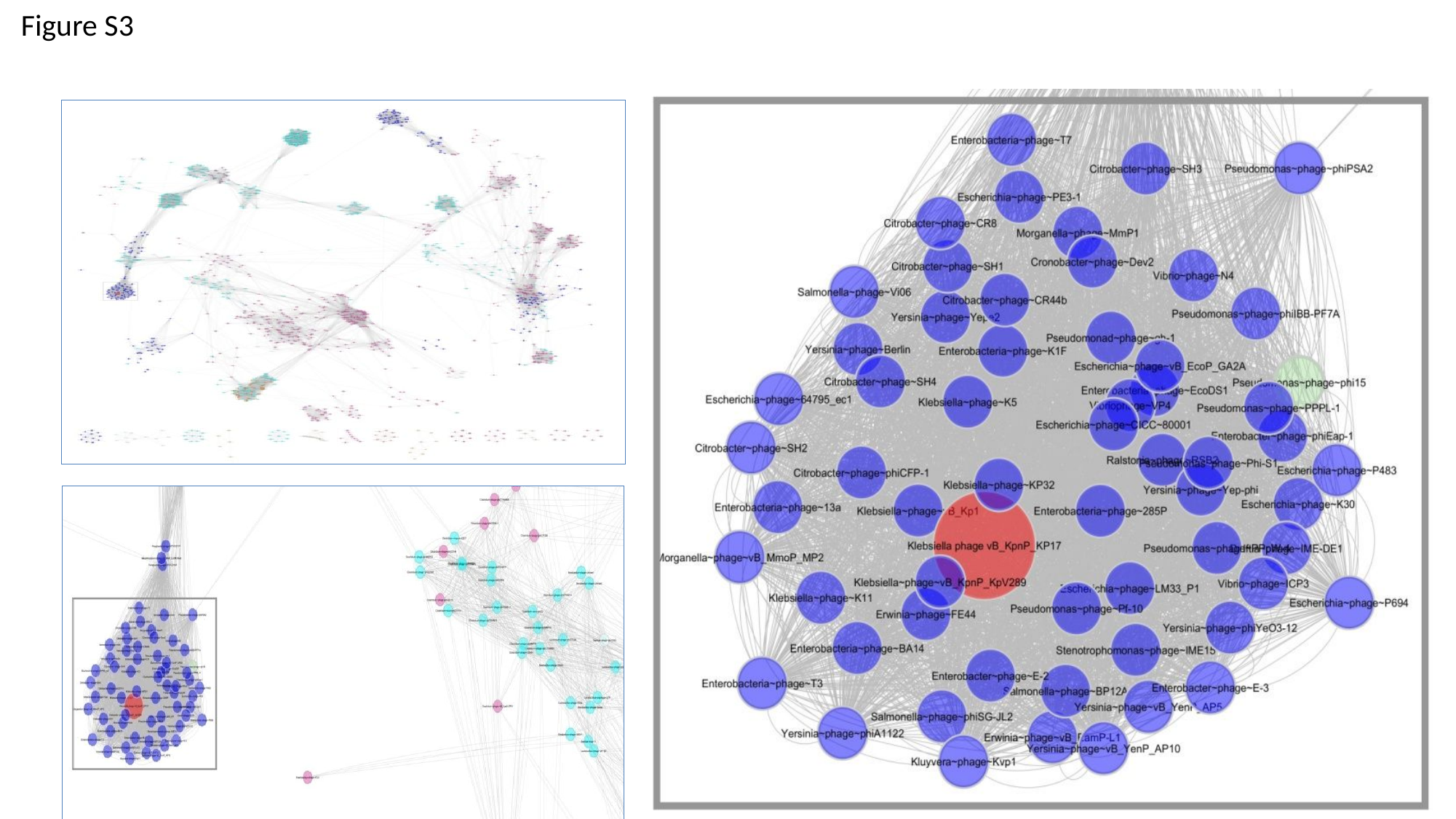

Figure S3

## Slide 4
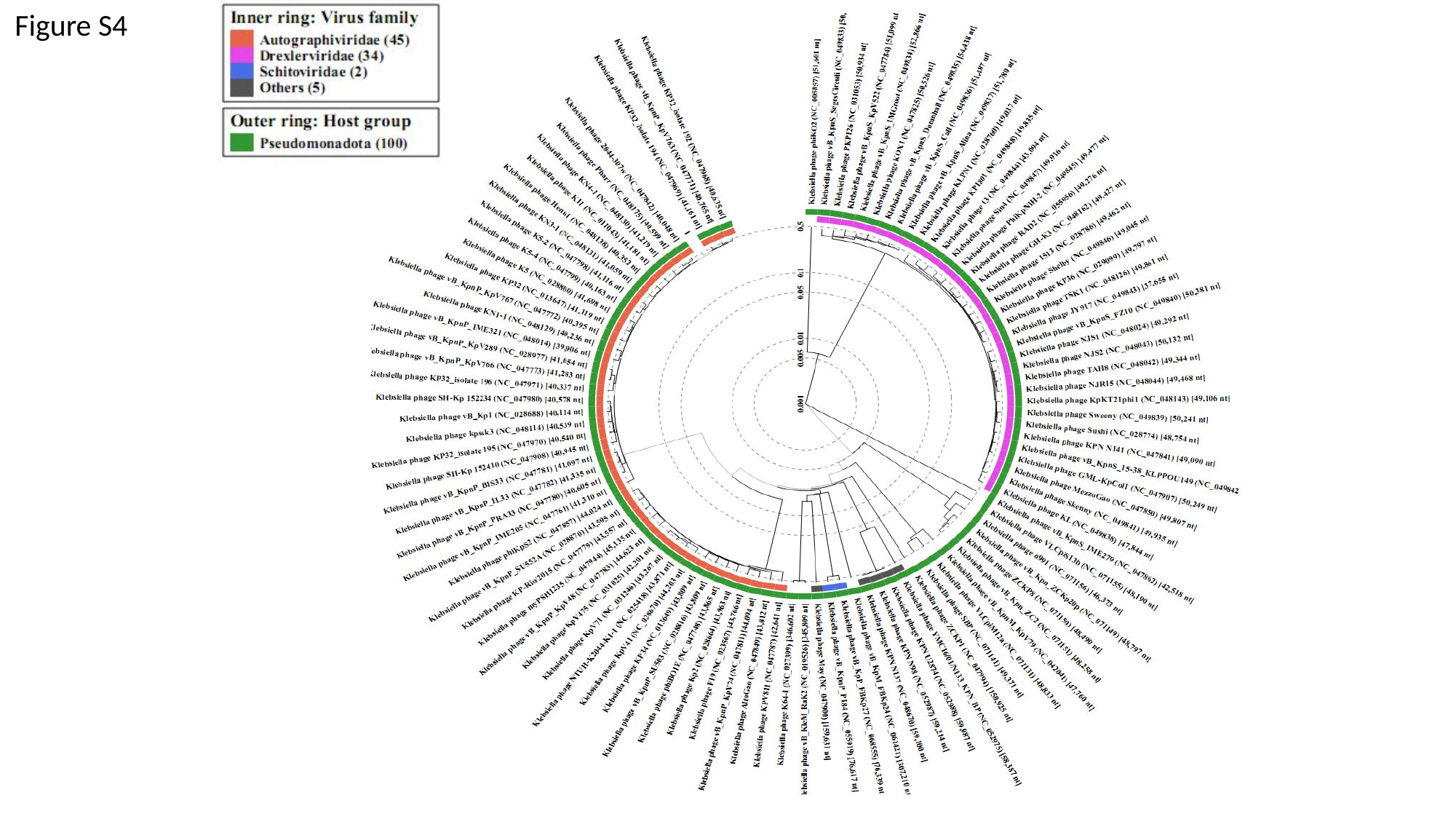

Figure S4
