# Supplementary material for: Analysis of a novel phage as a promising biological agent targeting multidrug resistant Klebsiella pneumoniae
Source: AMB Express. 2025 Mar 5;15:37. doi: 10.1186/s13568-025-01846-0 (PMC11882492; doi:10.1186/s13568-025-01846-0)
Supplement: Supplementary file 2 — Supplementary Material 2 (DOCX 40 KB) [file 13568_2025_1846_MOESM2_ESM.docx]

**Supplementary table S1: Predicted ORFs identified in *Klebsiella* phage vB_KpnP_KP17**

| Coding  Sequence | Start | Stop | Strand | GC  (%) | Protein length | MW (KDa) | Gene name | Putative function | Amino acid sequence similarity  to best homologs | Accession no |
| --- | --- | --- | --- | --- | --- | --- | --- | --- | --- | --- |
| ORF1 | 814 | 1281 | + | 50.64 | 156 | **17.37** | S-adenosyl-L-methionine hydrolase | Host cell lysis | *Klebsiella* phage IME264 | UMO77070.1 |
| ORF2 | 1281 | 1463 | + | 53.55 | 61 | **7** | Hypothetical protein | Unknown | *Klebsiella* phage vB_KpnP_IME321 | YP_009806211.1 |
| ORF3 | 1441 | 1638 | + | 48.99 | 66 | **7.62** | Hypothetical protein | Unknown | *Klebsiella* phage VLCpiA3c | UVX31353.1 |
| ORF4 | 1884 | 2765 | + | 54.76 | 294 | **33.52** | Serine/threonine kinase | DNA metabolism regulation | *Klebsiella* phage IME264 | UMO77066.1 |
| ORF5 | 2957 | 5677 | + | 55.68 | 907 | **101.53** | DNA dependent RNA polymerase | DNA replication, transcription, nucleic acids processing | *Klebsiella* phage NK20 | UUU45379.1 |
| ORF6 | 5776 | 6339 | + | 52.13 | 188 | **21.62** | Hypothetical protein | Unknown | *Klebsiella* phage vB_KpnP_K2044-EW | UZT48326.1 |
| ORF7 | 6423 | 6599 | + | 51.41 | 59 | **6.65** | Hypothetical protein | Unknown | *Klebsiella* phage K5-4 | YP_009788604.1 |
| ORF8 | 6960 | 7988 | + | 53.94 | 343 | **38.75** | DNA ligase | Replication, transcription, nucleic acids processing | *Klebsiella* phage vB_Kp168 | QGF21400.1 |
| ORF9 | 8106 | 8369 | + | 56.44 | 88 | **10.23** | BC10 family protein | Unknown | *Klebsiella* phage vB_KpnP_KpV289 | YP_009215465.1 |
| ORF10 | 8362 | 8793 | + | 52.78 | 144 | **16.07** | Nucleotide kinase | Replication, transcription, nucleic acids processing | *Klebsiella* phage vB_KpnP_KpV763 | YP_009786755.1 |
| ORF11 | 8780 | 8905 | + | 51.59 | 42 | **5.03** | Hypothetical protein | Unknown | *Klebsiella* phage vB_Kp1 | YP_009190966.1 |
| ORF12 | 8871 | 9020 | + | 55.33 | 50 | **5.43** | RNA polymerase inhibitor | Replication, transcription, nucleic acids processing | *Klebsiella* phage  KP32_isolate 192 | YP_009801318.1 |
| ORF13 | 9080 | 9775 | + | 53.59 | 232 | **25.6** | Gp2.5-like ssDNA binding protein and ssDNA annealing protein | Replication, transcription, nucleic acids processing  DNA replication and repair | *Klebsiella* phage vB_KpnP_BIS33 | YP_009787466.1 |
| ORF14 | 9747 | 10223 | + | 51.99 | 159 | **18.38** | Endonuclease I | Replication, transcription, nucleic acids processing | *Klebsiella* phage KMI1 | QDK04485.1 |
| ORF15 | 10226 | 10585 | + | 55.00 | 120 | **13.2** | Endolysin;  N-acetylmuramoyl-L-alanine amidase | Lysis | *Klebsiella* phage KMI2 | QEG09962.1 |
| ORF16 | 11128 | 12678 | + | 53.51 | 517 | **56.46** | DNA primase/helicase | Replication, transcription, nucleic acids processing | *Klebsiella* phage vB_Kpn_K80PH1317b | CAK1257371.1 |
| ORF17 | 12772 | 12981 | + | 50.00 | 70 | **7.4** | Hypothetical protein | Unknown | *Klebsiella* phage  vB_KpnP-VAC71 | UEP19686.1 |
| ORF18 | 12982 | 13164 | + | 50.27 | 61 | **6.65** | Hypothetical protein | Unknown | *Klebsiella* phage vB_KpnP_IME335 | QEQ50466.1 |
| ORF19 | 13177 | 13488 | + | 56.41 | 104 | **11.35** | Inhibitor | Replication, transcription, nucleic acids processing | *Klebsiella* phage P55 | WNO29417.1 |
| ORF20 | 13561 | 13953 | + | 53.44 | 131 | **14.63** | Hypothetical protein | Unknown | *Klebsiella* phage IME264 | UMO77048.1 |
| ORF21 | 13970 | 16096 | + | 53.97 | 709 | **80.1** | DNA polymerase I | Replication, transcription, nucleic acids processing | *Klebsiella* phage KN4-1 | YP_009817993.1 |
| ORF22 | 16115 | 16399 | + | 51.58 | 95 | **10.42** | HNS binding protein | DNA replication and transcription | *Klebsiella* phage Kp_Pokalde_002 | QKE60362.1 |
| ORF23 | 16396 | 16605 | + | 54.29 | 70 | **7.24** | HNS binding protein | DNA replication and transcription, DNA metabolism | *Klebsiella* phage vB_Kp1 | YP_009190979.1 |
| ORF24 | 16602 | 17507 | + | 54.42 | 301 | **34.22** | RNaseH | DNA metabolism regulation | *Klebsiella* phage VLCpiA3b | UVX30482.1 |
| ORF25 | 17489 | 17599 | + | 50.45 | 37 | **3.99** | Hypothetical protein | Unknown | *Klebsiella* phage vB_KpnP_KpV767 | YP_009786824.1 |
| ORF26 | 17686 | 17931 | + | 51.22 | 82 | **9.45** | Hypothetical protein | Unknown | *Klebsiella* phage TUN1 | CAF0683051.1 |
| ORF27 | 17934 | 18155 | + | 50.90 | 74 | **7.65** | DUF5476 domain-containing protein | Structural; capsid morphogenesis | *Klebsiella* phage vB_KpnP_KpV763 | YP_009786775.1 |
| ORF28 | 18157 | 18417 | + | 54.79 | 83 | **8.38** | Host range and adsorption protein | DNA metabolism regulation | *Klebsiella* phage K11 | YP_002003820.1 |
| ORF29 | 18414 | 18824 | + | 47.69 | 137 | **15.07** | HNH homing endonuclease | DNA packaging, replication, transcription, nucleic acids processing | *Klebsiella* phage 117 | QGH73702.1 |
| ORF30 | 18821 | 20431 | + | 55.00 | 537 | **58.76** | Head-tail adaptor | Structural; capsid morphogenesis | *Klebsiella* phage vB_Kpn_K80PH1317b | CAK1257468.1 |
| ORF31 | 20535 | 21497 | + | 53.06 | 321 | **35.54** | Capsid and scaffold protein | Structural; capsid morphogenesis | *Klebsiella* phage Kund-ULIP47 | QAU05536.1 |
| ORF32 | 21632 | 22663 | + | 57.07 | 344 | **36.41** | Major head protein | Structural; capsid  morphogenesis | *Klebsiella* phage  KP32_isolate 196 | YP_009801464.1 |
| ORF33 | 22720 | 22941 | + | 50.90 | 74 | **7.81** | Hypothetical protein | Unknown | *Klebsiella* phage vB_KpnP_KpV767 | YP_009786831.1 |
| ORF34 | 23009 | 23587 | + | 54.75 | 193 | **21.32** | Tail tubular  protein A | Structural; tail  morphogenesis | *Klebsiella* phage KP32 | 5MU4_A |
| ORF35 | 23610 | 23987 | + | 52.65 | 126 | **13.65** | Hypothetical protein | Unknown | *Klebsiella* phage 066024 | QOV06634.1 |
| ORF36 | 23954 | 25984 | + | 53.32 | 677 | **75.73** | Tail protein | Structural; tail morphogenesis | *Klebsiella* phage  KP32_isolate 194 | YP_009801379.1 |
| ORF37 | 26059 | 26469 | + | 48.18 | 137 | **15.87** | Internal (core) protein | Structural & morphogenesis | *Klebsiella* phage Kund-ULIP54 | QBG78381.1 |
| ORF38 | 26472 | 27062 | + | 53.13 | 197 | **21.05** | Internal virion protein | Structural, tail morphogenesis, DNA assembly | *Klebsiella* phage  KP32_isolate 192 | YP_009801339.1 |
| ORF39 | 27062 | 29317 | + | 55.63 | 752 | **84.67** | Internal (core) protein | Structural & morphogenesis | *Klebsiella* phage vB_Kp_IME531 | UEP18937.1 |
| ORF40 | 29292 | 29399 | - | 44.44 | 36 | **4.02** | Hypothetical protein | Unknown | *Klebsiella* phage 066042 | QOV07304.1 |
| ORF41 | 29744 | 33298 | + | 55.56 | 1185 | **127.81** | Internal (core) protein | Structural & morphogenesis | *Klebsiella* phage vB_Kp_IME531 | UEP18938.1 |
| ORF42 | 33359 | 33793 | + | 53.56 | 145 | **16.28** | Tail fiber protein | Structural; tail fiber morphogenesis | *Klebsiella* phage vB_KpnP_FZ12 | QCG76439.1 |
| ORF43 | 33790 | 34233 | + | 47.75 | 148 | **15.92** | Tail fiber protein | Structural; tail fiber morphogenesis | *Klebsiella* phage vB_KpnP_FZ12 | QCG76439.1 |
| ORF44 | 34262 | 35836 | + | 52.06 | 525 | **55.82** | Type II tail fiber/depolymerase | Structural; tail morphogenesis | *Klebsiella* phage Kp11 | UPO42307.1 |
| ORF45 | 35849 | 36064 | + | 50.46 | 72 | **7.74** | Holin | Host cell wall  lysis | *Klebsiella* phage vB_Kpn_K82P1 | CAK6605459.1 |
| ORF46 | 36055 | 36312 | + | 51.16 | 86 | **9.62** | Terminase small subunit | DNA genome Packaging | *Klebsiella* phage vB_KpnP_KpV767 | YP_009786841.1 |
| ORF47 | 36409 | 36855 | + | 50.11 | 149 | **16.55** | Rz-like spanin | Host cell wall  lysis | *Klebsiella* phage  KP32_isolate 194 | YP_009801388.1 |
| ORF48 | 36852 | 38057 | + | 52.82 | 402 | **45.07** | Terminase large subunit | DNA genome packaging | *Klebsiella* phage vB_Kpn_K37PH164C1 | CAK6596327.1 |
| ORF49 | 38026 | 38607 | + | 54.12 | 190 | **21.76** | Terminase large subunit | Packaging | *Klebsiella* phage vB_Kpl_K8PH128 | CAK6589044.1 |
| ORF50 | 38852 | 39001 | + | 51.33 | 50 | **5.38** | Hypothetical protein | Unknown | *Klebsiella* phage vB_KpnP_K2044-HW | UZT48231.1 |
