# Supplementary material for: Analysis of a novel phage as a promising biological agent targeting multidrug resistant Klebsiella pneumoniae
Source: AMB Express. 2025 Mar 5;15:37. doi: 10.1186/s13568-025-01846-0 (PMC11882492; doi:10.1186/s13568-025-01846-0)
Supplement: Supplementary file 3 — Supplementary Material 3 (DOCX 15 KB) [file 13568_2025_1846_MOESM3_ESM.docx]

**Supplementary table S2: Homology of *Klebsiella* phage vB_KpnP_KP17 to other *Klebsiella* phages genomes**

| Scientific name |  | Query coverage (%) | E value | Percent identity (%) | Accession length (bp) | Accession no |
| --- | --- | --- | --- | --- | --- | --- |
| *Klebsiella* phage KP32 |  | 93% | 0 | 94.79 | 40635 | NC_047968.1 |
| *Klebsiella* phage vB_Kpn_K37PH164C1 |  | 93% | 0 | 94.71 | 40674 | OY978834.1 |
| *Klebsiella* phage Kp9 |  | 93% | 0 | 93.97 | 40337 | ON148529.1 |
| *Klebsiella* phage vB_KpnP_KpV763 |  | 93% | 0 | 93.88 | 40765 | NC_047771.1 |
| *Klebsiella* phage IME183 |  | 94% | 0 | 93.81 | 41384 | MZ398245.3 |
| *Klebsiella* phage vB_KpnP_EKp2 |  | 93% | 0 | 93.71 | 39320 | OQ921102.1 |
| *Klebsiella* phage cp7 |  | 94% | 0 | 93.3 | 40519 | OX335390.1 |
| *Klebsiella* phage Kp11 |  | 94% | 0 | 93.28 | 39935 | ON148528.1 |
